# Heterologous Production, Purification and Characterization of Two Cold-Active β-d-Galactosidases with Transglycosylation Activity from the Psychrotolerant Arctic Bacterium *Arthrobacter* sp. S3* Isolated from Spitsbergen Island Soil

**DOI:** 10.3390/ijms252413354

**Published:** 2024-12-12

**Authors:** Marta Wanarska, Anna Pawlak-Szukalska, Aleksandra Rosińska, Katarzyna Kozłowska-Tylingo

**Affiliations:** 1 Department of Biotechnology and Microbiology, Faculty of Chemistry, Gdansk University of Technology, Narutowicza 11/12, 80-233 Gdansk, Poland; a.pawlak.szukalska@gmail.com (A.P.-S.); rosinska.aleks@gmail.com (A.R.); 2 Department of Pharmaceutical Technology and Biochemistry, Faculty of Chemistry, Gdansk University of Technology, Narutowicza 11/12, 80-233 Gdansk, Poland; katarzyna.kozlowska-tylingo@pg.edu.pl

**Keywords:** cold-active enzyme, β-d-galactosidase, *Arthrobacter* sp., Arctic soil, lactose hydrolysis, transglycosylation

## Abstract

Cold-adapted microorganisms possess cold-active enzymes with potential applications in different industries and research areas. In this study, two genes encoding β-d-galactosidases belonging to Glycoside Hydrolase families 2 and 42 from the psychrotolerant Arctic bacterium *Arthrobacter* sp. S3* were cloned, expressed in *Escherichia coli* and *Komagataella phaffii*, purified and characterized. The GH2 β-d-galactosidase is a tetramer with a molecular weight of 450 kDa, while the GH42 β-d-galactosidase is a 233 kDa trimer. The Bgal2 was optimally active at pH 7.5 and 22 °C and maintained 57% of maximum activity at 10 °C, whereas the Bgal42 was optimally active at pH 7.0 and 40 °C and exhibited 44% of maximum activity at 10 °C. Both enzymes hydrolyzed lactose and showed transglycosylation activity. We also found that 2 U/mL of the Bgal2 hydrolyzed 85% of lactose in milk within 10 h at 10 °C. The enzyme synthesized galactooligosaccharides, heterooligosaccharides, alkyl galactopyranosides and glycosylated salicin. The Bgal42 synthesized galactooligosaccharides and 20 U/mL of the enzyme hydrolyzed 72% of milk lactose within 24 h at 10 °C. The properties of *Arthrobacter* sp. S3* Bgal2 make it a candidate for lactose hydrolysis in the dairy industry and a promising tool for the glycosylation of various acceptors in the biomedical sector.

## 1. Introduction

Biocatalysts are widely used across various industrial sectors, including food and beverages, detergents, animal feed, pharmaceuticals, biofuel, textiles, pulp and paper, personal care and cosmetics, wastewater and agriculture [1]. The global industrial enzymes market size was estimated to be USD 7.53 billion in 2024 and is expected to grow at a compound annual growth rate (CAGR) of 6.4% from 2024 to 2030 [2]. The continuous growth in the industrial enzymes market is driven by increasing global population and, consequently, the demand for consumer goods. Additionally, growing awareness of green technologies and the need to address environmental challenges are significantly promoting the utilization of enzymes in the commercial production of these goods. The highest share in revenue at the level of 48.34% in 2023 was achieved by the market of glycoside hydrolases [2]. Enzymes derived from microorganisms, fungi, bacteria and yeasts dominate the market because they provide high activity, high yields, reproducibility, cost reductions, and simplicity [1]. Microorganisms inhabiting cold environments (psychrophiles and psychrotolerants), among other features, produce enzymes with more flexible structures compared to their mesophilic counterparts, which enables their activity at low temperatures and also facilitates thermal inactivation. The high activity of psychrozymes at low temperatures contributes to energy savings and enables the performance of technological processes requiring heat-sensitive and volatile components, in which undesirable side-reactions or contamination problems must be avoided. Moreover, cold-active enzymes can operate at low water activity, which allows the use of high substrate concentrations or the addition of organic solvents to the reaction mixtures [3,4,5].

Extremozymes, including cold-active enzymes, are robust biocatalytic tools, but numerous limitations associated with the cultivation of extremophiles, such as the use of high/low-temperature incubators, culture vessels resistant to corrosion caused by high acidity/alkalinity/salinity, specific media, and long incubation times, necessitate the use of heterologous hosts, most often mesophilic *Escherichia coli* bacterium or *Komagataella phaffii* (formerly *Pichia pastoris*) yeast, for the efficient and cost-effective production of extremozymes [4,6,7]. Additionally, protein engineering can be used to improve the properties of recombinant biocatalysts [4,8].

Among the enzymes used in the food and beverage industry, β-d-galactosidase (lactase) is particularly important. This glycoside hydrolase (GH) catalyzes the hydrolysis of glycosidic linkages in β-d-galactosides, and the best-known and abundant natural substrate of β-d-galactosidase is milk sugar lactose. The main industrial application of lactase is the production of milk and other dairy products with a reduced content of lactose or no lactose for people suffering from lactose intolerance. Another application of β-d-galactosidase is the processing of dairy industry by-products, cheese whey and whey permeate. The hydrolysis of lactose into the monosaccharides glucose and galactose makes them sweeter and more digestible, so they can be used as additives in food, beverages and animal feed [3,8]. The β-d-galactosidase has also been applied in biosensor assembly to exactly quantify the lactose in dairy products [9]. In addition, some β-d-galactosidases catalyze the transfer of a galactosyl moiety from a sugar donor, mostly lactose, to an acceptor, resulting in the synthesis of various oligosaccharides and other glycoconjugates for the food, cosmetic and pharmaceutical industries [3,10]. The best-known products of lactose transgalactosylation catalyzed by β-d-galactosidase are galactooligosaccharides (GOS), which are sugars composed of up to 10 glucose and galactose residues with prebiotic properties. Prebiotics are defined as nondigestible, selectively fermented ingredients that enable specific changes in the composition and/or activity of the gastrointestinal microbiota that confer benefits upon the health and well-being of the host [8,11]. For example, GOS supplementation has been shown to reduce stress-induced gastrointestinal dysfunction in healthy college students, as well as shorten the duration of colds and flu [12]. Due to many well-proven health-promoting properties, GOS are used as ingredients in functional foods and beverages [8,11]. Moreover, the addition of probiotic bacteria from *Bifidobacterium* or *Lactobacillus* genera and prebiotics such as GOS and FOS (fructooligosaccharides) to infant formulas allows at least some mimicking of human milk [13]. GOS have also been investigated as potential dietary supplements for pregnant women [14]. For these reasons, the demand for GOS is constantly growing. The global GOS market size was USD 570 million in 2021 and is expected to grow at a CAGR of 6.5% from 2022 to 2029 [15].

Lactulose, a nondigestible disaccharide used as a medicinal drug to treat constipation and hepatic encephalopathy and as a prebiotic in functional food, can be produced by the β-d-galactosidase-catalyzed transgalactosylation of fructose [16,17]. Moreover, the β-d-galactosidase from *Bacillus circulans* was successfully applied as biocatalyst for the production of lactosucrose trisaccharide from lactose and sucrose by transgalactosylation [18,19]. In Japan, lactosucrose is widely used in healthy foods and drinks due to its prebiotic properties [20]. The biosynthesis of alkyl glycosides, which can be used in the detergent and cosmetic industries as nonionic surfactants, as well as in the biosynthesis of glycosylated anti-microbial and anti-cancer drugs for pharmaceutical applications, has also been performed using β-d-galactosidases [10]. Hence, the global β-d-galactosidase market was valued at USD 193.6 million in 2020 and was projected to grow at a CAGR of 5.1% from 2020 to 2027. The lactase market volume was 505.9 tons in 2020, and the forecast volume is 700.0 tons in 2027. It is worth mentioning that in 2019, Europe was the leader in the lactase market and accounted for over 48% of global revenues, due to the presence of key manufacturers such as DSM and Chr. Hansen (currently a part of Novonesis Group, after merging with Novozymes), a developed food and beverage industry, and growing health and environmental awareness [21]. Currently, commercial preparations of β-d-galactosidase are derived from mesophilic microorganisms, namely *Kluyveromyces* spp. yeasts, for the production of low-lactose milk and other dairy products; dietary supplement products (44% share of the global revenue in 2019); *Aspergillus* spp. filamentous fungi for lactose hydrolysis in acidic whey [21]; and *B. circulans* bacterium for the synthesis of prebiotic oligosaccharides [22]. However, their counterparts originating from cold-adapted microbes still have great application potential. To the best of our knowledge, only one cold-active β-d-galactosidase, which is produced by the psychrotolerant yeast *Guehomyces pullulans*, is used as an industrial enzyme [21].

In this context, the aim of this study was to produce two novel β-d-galactosidases originating from a psychrotolerant bacterium isolated from an Arctic soil sample and to investigate their catalytic potential with respect to hydrolysis and transglycosylation, especially at low temperatures.

## 2. Results

### 2.1. Isolation, Identification and General Characterization of a Bacterial Strain S3*

Fifty one bacterial isolates were obtained from a soil sample collected in the neighborhood of the Polish polar station at Isbjørnhamna in Hornsund fjord on Spitsbergen island, in the Norwegian Svalbard archipelago (77°00′ N, 15°33′ E). All isolates have been cryopreserved as glycerol stocks at −80 °C and deposited in the local microbial culture collection at Gdańsk University of Technology (GUT). Five of them showed β-d-galactosidase activity on agar medium supplemented with X-gal and IPTG. Due to the highest β-d-galactosidase activity, the isolate named S3* was selected for further studies. It did not exhibit other enzymatic activities such as proteolytic, amylolytic, esterolytic and lipolytic activities. The bacterial colonies formed on the agar medium were circular, smooth, shiny and pale yellow in color. The optimal aerobic growth of S3* isolate was observed at 22 to 24 °C. The growth was weak at 30 °C and no growth occurred at 37 °C. Hence, the S3* isolate can be categorized as a psychrotolerant bacterium. The cells of the S3* isolate were Gram positive and displayed a rod–coccus morphological cycle (rod shape in young culture and coccus shape in old culture).

A comparison of the 16S rRNA gene partial sequence of the S3* isolate (GenBank, accession no. PQ463689) with those available in the GenBank database at NCBI using the BLASTN 2.16.1 program [23] indicated that it belongs to the *Arthrobacter* genus, and that its closest relatives are *Arthrobacter glacialis* strain HLT2-12-2 (GenBank, accession no. NR_178556.1) and *Arthrobacter alpinus* strain S6-3 (GenBank, accession no. NR_117254.1), with 100% query coverage and 98.90 and 98.29% sequence identity, respectively.

### 2.2. Construction of an Arthrobacter sp. S3* Genomic Library and Identification of β-d-Galactosidase Genes

The *Arthrobacter* sp. S3* genomic DNA library was prepared in the β-d-galactosidase-deficient *E. coli* strain TOP10F’. Three recombinant bacterial colonies, among approximately 4000, exhibited the activity of heterologous β-d-galactosidase on Luria–Bertani (LB) agar plates supplemented with X-gal and IPTG. The restriction analysis of pBAD/β-galS3* Lib1, pBAD/β-galS3* Lib2 and pBAD/β-galS3* Lib3 plasmids showed that they contained *Arthrobacter* sp. S3* genomic DNA fragments of about 26–28, 19–20 and 38–40 kbp, respectively. The genomic DNA inserts of the pBAD/β-galS3* Lib1 and Lib2 plasmids were partially sequenced.

The analysis of the pBAD/β-galS3* Lib1 insert sequence revealed an open reading frame consisting of 3090 bp (GenBank, accession no. PQ479351), which displayed only four nucleotide differences from the *Arthrobacter* sp. ON14 *galA* gene (GenBank Accession no. HM178943.1), namely 246 T/G, 485 G/N, 2015 C/T and 2614 A/C, and there was one undefined nucleotide at position 485 in the *galA* gene. The obtained ORF encoded a 1029 amino acid protein with a calculated molecular mass of 111,598.3 Da and a theoretical pI of 4.95 (ProtParam program on the ExPASy Proteomics Server) [24]. A homology search performed using NCBI’s protein–protein version of BLAST (BLASTP 2.16.1) [25] showed 1026/1029 amino acid identities and 1027/1029 similarities with GalA β-d-galactosidase from *Arthrobacter* sp. ON14. There were three amino acid alterations, namely G162X, P672L and M872L, among which X encoded by the GNC codon can be A, D, G or V. According to the FASTA 1 program at the EMBL’s European Bioinformatics Institute (EMBL-EBI) [26], the *Arthrobacter* sp. S3* β-d-galactosidase also showed high homology with other Glycoside Hydrolase family 2 β-d-galactosidases from *Arthrobacter* spp. (Table 1). A conserved domain search performed using the InterPro 98.0 database at the EMBL-EBI [27], found four conserved domains in this *Arthrobacter* sp. S3* β-d-galactosidase: a Glycoside Hydrolase family 2 sugar-binding domain (Glyco_hydro_2_N, residues 34-218), a Glycoside Hydrolase family 2 TIM barrel domain (Glyco_hydro_2_C, catalytic domain, residues 319-618), a β-galactosidase domain 4 (LacZ_4, residues 631-721), and a β-galactosidase small-chain/domain 5 (Bgal_small_N_2, residues 744-1027).

The analysis of the pBAD/β-galS3* Lib2 insert sequence revealed an open reading frame consisting of 2052 bp (GenBank, accession no. PQ479352), which displayed only three nucleotide differences from the *Arthrobacter* sp. ON14 *galB* gene (GenBank, accession no. HM178942.1), namely 1162 A/G, 1968 C/T and 1994 A/G. This ORF encoded a 683 amino acid protein with a calculated molecular mass of 74,390.4 Da and a theoretical pI of 5.00 (ProtParam program on the ExPASy Proteomics Server [24]). A homology search, performed using NCBI’s BLASTP 2.16.1 [25], showed 681/683 amino acid identities with GalB β-d-galactosidase from *Arthrobacter* sp. ON14 (GenBank, accession no. ADJ18282.1). There were two amino acid alterations, namely S388/G and D665/G. An additional homology search performed using the EMBL-EBI’s FASTA 1 program [26] displayed high amino acid identity and similarity with β-d-galactosidase from *Arthrobacter* sp. 32c, and some homology with other cold-active β-d-galactosidases belonging to the Glycoside Hydrolase family 42 (Table 2). Moreover, three conserved domains were found in this *Arthrobacter* sp. S3* protein using the InterPro 98.0 database [27], namely a Glycoside Hydrolase family 42 N-terminal (Glyco_hydro_42_N, residues 36-407), a β-galactosidase trimerization domain (Glyco_hydro_42M, residues 419-619), and a β-galactosidase C-terminal domain (Glyco_hydro_42C, residues 628-679).

### 2.3. Production and Purification of Arthrobacter sp. S3* Bgal2 and Bgal42 β-d-Galactosidases

**Figure 1:**
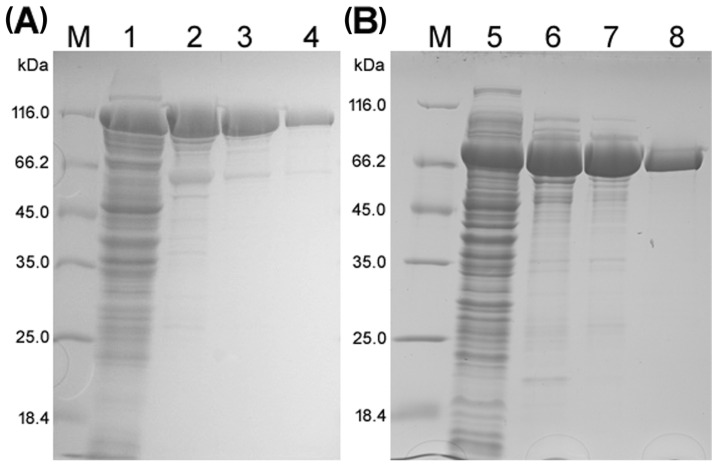
SDS-PAGE analysis of the fractions obtained by expression and purification of *Arthrobacter* sp. S3* Bgal2 (**A**) and Bgal42 (**B**) β-d-galactosidases. Lane M—Unstained Protein Molecular Weight Marker (Thermo Fisher Scientific Baltics, Vilnius, Lithuania): 116.0, 66.2, 45.0, 35.0, 25.0, 18.4 and 14.4 kDa; lane 1—cell-free extract of *E. coli* LMG194 after *bgal2* gene expression; lane 2—purified Bgal2 protein after ion exchange chromatography on Fractogel EMD DEAE column; lane 3—purified Bgal2 after ion exchange chromatography on Fractogel EMD TMAE column; lane 4—purified Bgal2 β-d-galactosidase after gel filtration; lane 5—cell-free extract of *E. coli* LMG194 after *bgal42* gene expression; lane 6—purified Bgal42 protein after IEX on Fractogel EMD DEAE column; lane 7—purified Bgal42 after IEX on Fractogel EMD TMAE column; lane 8—purified Bgal42 β-d-galactosidase after GF.

In order to produce and investigate the biochemical properties of *Arthrobacter* sp. S3* Bgal2 and Bgal42 enzymes, two recombinant plasmids named pBAD/Myc-HisA-GH2β-galS3* and pBAD-GH42BgalS3* were constructed and used for the expression of *bgal2* and *bgal42* genes in the *lac*Z-deficient *E. coli* LMG194 strain at 30 °C. Recombinant enzymes were purified by ion exchange chromatography (IEX), using weak and strong anion exchangers, and by gel filtration (GF). After purification, SDS-PAGE, and staining with Coomassie Brilliant Blue, the predominant protein bands migrating near 116.0 and slightly above 66.2 kDa were observed for Bgal2 and Bgal42 (Figure 1A,B), respectively. These results were consistent with the calculated molecular weights of Bgal2 and Bgal42 β-d-galactosidases of *Arthrobacter* sp. S3*, namely 111.6 and 74.4 kDa.

The expression system based on the l-arabinose inducible *E. coli araBAD* promoter and purification method applied were quite efficient, yielding 11 mg of recombinant *Arthrobacter* sp. S3* GH2 β-d-galactosidase with a specific activity of 86.9 U mg^−1^ and 20 mg of recombinant GH42 β-d-galactosidase with a specific activity of 115 U mg^−1^ from 1 L of *E. coli* LMG194 culture (Table 3).

The molecular weights of native Bgal2 and Bgal42 enzymes, as estimated by GF, were 450 and 233 kDa, respectively. These data suggest that Bgal42 is a trimeric protein, while Bgal2 forms a tetramer in the solution.

The *bgal*2 and *bgal*42 genes were also expressed in the methylotrophic yeast *Komagataella phaffii* (formerly *Pichia pastoris*) strain X-33 under the control of a methanol-inducible *AOX1* promoter (P_AOX1_). Both *Arthrobacter* sp. S3* β-d-galactosidases were expressed as fusions to the *Saccharomyces cerevisiae* α-factor signal peptide, which was used to direct the secretion of recombinant proteins. After 4 days of recombinant *K. phaffii* strain cultivation in the medium supplemented with methanol, 465 U of the Bgal42 enzyme was obtained per 1 L of post-culture fluid. No Bgal2 enzyme activity was detected in the post-culture medium of the recombinant *K. phaffii* strain harboring the *bgal*2 gene. Furthermore, the activity of both enzymes in the cell-free extracts of recombinant yeast strains was determined and was 530 and 474 U per 1 L of culture for Bgal42 and Bgal2, respectively. After 5 days of recombinant yeast strain cultivation, the activity of both *Arthrobacter* sp. S3* β-d-galactosidases was only slightly higher.

### 2.4. Structure Prediction of Arthrobacter sp. S3* Bgal2 and Bgal42 β-d-Galactosidases

**Figure 2:**
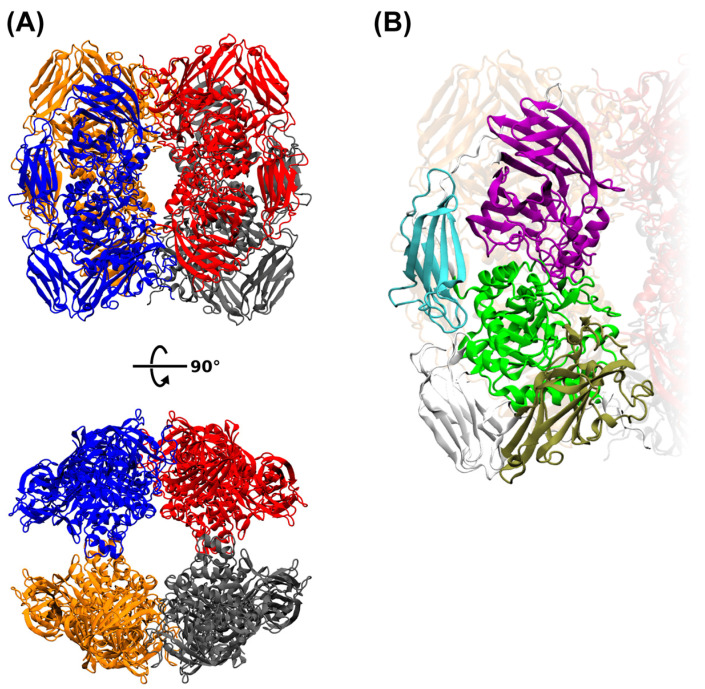
The 3D prediction of the *Arthrobacter* sp. S3* Bgal2 protein structure obtained with AlphaFold2. (**A**) Tetrameric structure of the Bgal2 β-d-galactosidase. Each monomer was colored with a different color: blue, red, orange and grey. (**B**) The predicted monomer of the Bgal2 was colored according to the conserved domains. The Glycoside Hydrolase family 2 sugar-binding domain (residues 34-218) was colored olive green, Glycoside Hydrolase family 2 TIM barrel domain (residues 319-618) was colored green, β-galactosidase domain 4 (residues 631-721) was colored light blue, and β-galactosidase small chain/domain 5 (residues 744-1027) was colored purple.

**Figure 3:**
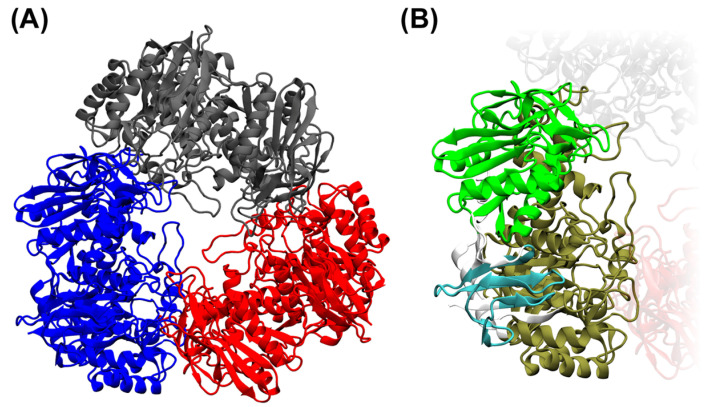
The 3D prediction of the *Arthrobacter* sp. S3* Bgal42 protein structure obtained with AlphaFold2. (**A**) Trimeric structure of the Bgal42 β-d-galactosidase. Each monomer was colored with a different color: blue, red and grey. (**B**) The predicted monomer of the Bgal42 was colored according to the conserved domains. The Glycoside Hydrolase family 42 N-terminal domain (residues 36-407) was colored olive green, β-galactosidase trimerization domain (residues 419-619) was colored green, and β-galactosidase C-terminal domain (residues 628-679) was colored light blue.

Protein structure prediction, carried out with the AlphaFold2 open-source code [28], was used to determine the three-dimensional shapes of the *Arthrobacter* sp. S3* Bgal2 and Bgal42 proteins from their amino acid sequences (Figure 2A,B and Figure 3A,B). The obtained structure predictions were compared with 3D structures from the RCSB Protein Data Bank, and the template modeling (TM) scores were calculated using the tmtools 0.2.0 python package [29]. The *Arthrobacter* sp. S3* Bgal2 was compared with the β-d-galactosidase from *Arthrobacter* sp. 32cB (RSCB PDB, accession no. 6SEB) [30], and the TM score was 0.925. The Bgal42 was compared with β-d-galactosidases from *T. thermophilus* A4 (RSCB PDB, accession no. 1KWG) [31] and *Rahnella* sp. R3 (RSCB PDB, accession no. 5E9A) [32], and the TM scores were 0.892 and 0.929, respectively.

### 2.5. Properties of Arthrobacter sp. S3* Bgal2 and Bgal42 β-d-Galactosidases

In order to determine the optimum pH of recombinant *Arthrobacter* sp. S3* Bgal2 and Bgal42 activity, the hydrolysis of synthetic substrate ONPG was performed at various pH values from 4 to 10 at room temperature (21–22 °C). The GH2 β-d-galactosidase had an apparent pH optimum of 7.5, and retained more than 88% of its maximum activity between pH 7.0 and 8.0. The GH42 β-d-galactosidase exhibited maximum activity at pH 7.0 and maintained at least 90% of maximum activity in a pH range of 6.0 to 8.0.

An investigation into the effect of temperature on the Bgal2 enzyme showed that the highest hydrolytic activity towards ONPG occurred at 22 °C in the 20 mM potassium phosphate buffer at pH 7.5 supplemented with 50 mM of KCl. The enzyme exhibited more than 90% of maximum activity in a temperature range of 20 to 25 °C, and maintained 39–57% of maximum activity at 5 to 10 °C. In the absence of KCl, the *Arthrobacter* sp. S3* GH2 β-d-galactosidase was unstable and rapidly lost activity. At 25 °C, over 60% of the initial activity of the enzyme was lost in an incubation period of 1 h and the *Arthrobacter* sp. S3* Bgal2 was inactivated within 5 min at 45 °C.

The hydrolytic activity of *Arthrobacter* sp. S3* Bgal42 towards ONPG was apparently optimal at 40 °C when assayed in the 20 mM potassium phosphate buffer at pH 7.0, and the enzyme retained more than 90% of maximum activity at temperatures ranging from 35 to 45 °C. Moreover, the Bgal42 exhibited 38–44% of maximum activity at 5 to 10 °C. The *Arthrobacter* sp. S3* GH2 β-d-galactosidase was stable at 40 °C, given that approximately 80% of its initial activity was retained after 2 h of incubation. At 43 °C, approximately 50% of initial activity was lost in an incubation period of 1 h and the enzyme was inactivated within 10 min at 50 °C.

To examine the metal ion requirements, the purified *Arthrobacter* sp. S3* β-d-galactosidases were assayed in the presence of EDTA. Bgal2 was strongly inhibited by this chelator of divalent cations, whereas the hydrolytic activity of Bgal42 was strongly enhanced in the presence of EDTA (Table 4). Moreover, both enzymes were strongly activated by Mg^2+^ and inhibited by Zn^2+^, Ni^2+^, Co^2+^ and Mn^2+^ ions. The *Arthrobacter* sp. S3* GH2 β-d-galactosidase was also partially inhibited by Ca^2+^ cations (by 45%). On the other hand, the activity of GH42 β-d-galactosidase was strongly enhanced in the presence of Ca^2+^ ions (Table 4). Furthermore, most of the tested reducing agents decreased the activity of both enzymes, with TCEP inhibiting it almost completely (Table 4).

The substrate specificity studies demonstrated that the *Arthrobacter* sp. S3* Bgal2 hydrolyzed only *p*-NP-β-d-galactopyranoside, and it exhibited less than 1% of the PNPG activity towards nine other substrates, namely *p*NP-β-d-glucopyranoside, *p*NP-β-d-fucopyranoside, *p*NP-β-d-mannopyranoside, *p*NP-β-d-xylopyranoside, *p*NP-β-l-arabinopyranoside, *p*NP-β-d-cellobioside, *p*NP-β-d-glucuronide, *p*NP-α-d-galactopyranoside and *p*NP-α-d-glucopyranoside. The *Arthrobacter* sp. S3* Bgal42 showed high activity towards PNPG and weak activity against *p*NP-β-d-fucopyranoside (about 5% of the PNPG activity).

The kinetic parameters of the synthetic substrate ONPG and natural substrate lactose hydrolysis catalyzed by the *Arthrobacter* sp. S3* GH42 β-d-galactosidase were determined at temperatures from 10 to 40 °C. The Bgal42 showed the highest catalytic efficiency (*k*_cat_/*K*_m_) towards lactose at 40 °C, but it was over two hundred times lower than that for ONPG. The *k*_cat_/*K*_m_ values for lactose were the same at 20 and 30 °C, and were two times higher than the catalytic efficiency determined at 10 °C (Table 5). The *Arthrobacter* sp. S3* GH42 β-d-galactosidase was inhibited by both d-glucose and d-galactose. The apparent *K*_i_ values determined at 30 °C using ONPG as the substrate were 2.99 and 6.01 mM for d-galactose and d-glucose, respectively. D-Galactose was a competitive inhibitor given that the apparent *V*_max_ value was almost unchanged, while the apparent *K*_m_ value increased from 1.23 to 4.01 mM. d-Glucose was an uncompetitive inhibitor because the apparent *V*_max_ value decreased from 276.94 to 164.25 U mg^−1^, and the apparent *K*_m_ value decreased from 1.23 to 0.67 mM.

The kinetic parameters of ONPG and the lactose hydrolysis catalyzed by the *Arthrobacter* sp. S3* GH2 β-d-galactosidase were determined at 10 and 20 °C. At 20 °C, the enzyme exhibited catalytic efficiency towards the synthetic substrate that was two orders of magnitude higher than that for the natural substrate (Table 5). Importantly, the activity of Bgal2 towards ONPG (5 mM) was not inhibited by either d-glucose or d-galactose in concentrations up to 100 mM. Moreover, the *k*_cat_/*K*_m_ value determined at 10 °C with the natural substrate lactose was six times higher for Bgal2 than Bgal42 (Table 5).

Experiments on the hydrolysis of lactose in commercially available UHT milk using *Arthrobacter* sp. S3* Bgal2 revealed that 85% of disaccharide was digested into d-glucose and d-galactose by 1 U of the β-d-galactosidase (11.5 µg of the protein) per 1 mL of milk over 24 h at 10 °C. During the incubation of 2 U Bgal2 (23.0 µg of the protein) in 1 mL of milk at 10 °C, 85% of the lactose was hydrolyzed within 10 h. The addition of 1 and 2 U of *Arthrobacter* sp. S3* Bgal42 (8.7 and 17.4 µg of the protein) to 1 mL of milk resulted in 12 and 18% yields of lactose hydrolysis after 24 h of incubation at 10 °C. However, 10 and 20 U of the enzyme (86.9 and 173.9 µg) digested 38 and 72% of milk disaccharide under the same conditions.

**Figure 4:**
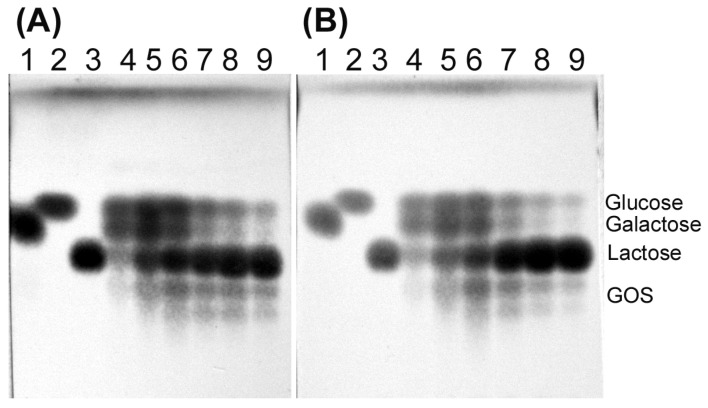
The TLC analysis of GOS synthesis catalyzed by the *Arthrobacter* sp. S3* Bgal2 enzyme. Reaction mixtures containing 2 U mL^−1^ of *Arthrobacter* sp. GH2 β-d-galactosidase and lactose as a substrate were incubated at 10 °C for 6 h (**A**) and at 20 °C for 8 h (**B**). Lane 1—d-galactose, lane 2—d-glucose, lane 3—lactose, lane 4—29 mM concentration of lactose in the reaction mixture, lane 5—58 mM concentration of lactose in the reaction mixture, lane 6—146 mM concentration of lactose in the reaction mixture, lane 7—292 mM concentration of lactose in the reaction mixture, lane 8—438 mM concentration of lactose in the reaction mixture, lane 9—584 mM concentration of lactose in the reaction mixture.

The transglycosylation activity of both *Arthrobacter* sp. S3* β-d-galactosidases was tested using various concentrations of lactose. The synthesis of galactooligosaccharides (GOS) was conducted at temperatures ranging from 10 to 40 °C. The Bgal2 exhibited high transglycosylation activity at 10 and 20 °C, and it was not active at 30 °C. The efficient synthesis of tri- and tetrasaccharides was achieved in 146–438 mM solutions of lactose containing 2 U mL^−1^ of the enzyme after 6–8 h of incubation, with the optimum being at 146 mM (Figure 4A,B).

**Figure 5:**
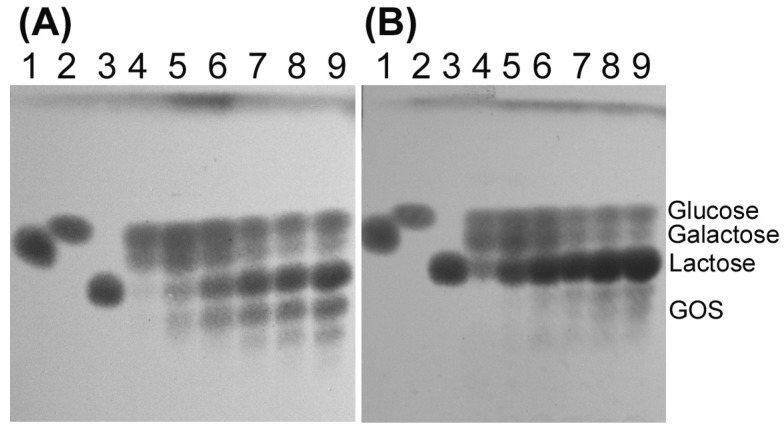
The TLC analysis of GOS synthesis catalyzed by the *Arthrobacter* sp. S3* Bgal42 enzyme. Reaction mixtures containing 10 U mL^−1^ of *Arthrobacter* sp. GH42 β-d-galactosidase and lactose as a substrate were incubated at 30 °C for 4 h (**A**) and at 40 °C for 8 h (**B**). Lane 1—d-galactose, lane 2—d-glucose, lane 3—lactose, lane 4—29 mM concentration of lactose in the reaction mixture, lane 5—58 mM concentration of lactose in the reaction mixture, lane 6—146 mM concentration of lactose in the reaction mixture, lane 7—292 mM concentration of lactose in the reaction mixture, lane 8—438 mM concentration of lactose in the reaction mixture, lane 9—584 mM concentration of lactose in the reaction mixture.

In the case of Bgal42, the highest yields of GOS (tri- and tetrasaccharides) synthesis were achieved after the incubation of reaction mixtures containing 292–548 mM of lactose and 10 U mL^−1^ of the enzyme at 30 °C for 4 h (Figure 5A). Bgal42 did not show any transglycosylation activity at 10 °C and its transferase activity at 40 °C was relatively low (Figure 5B).

**Figure 6:**
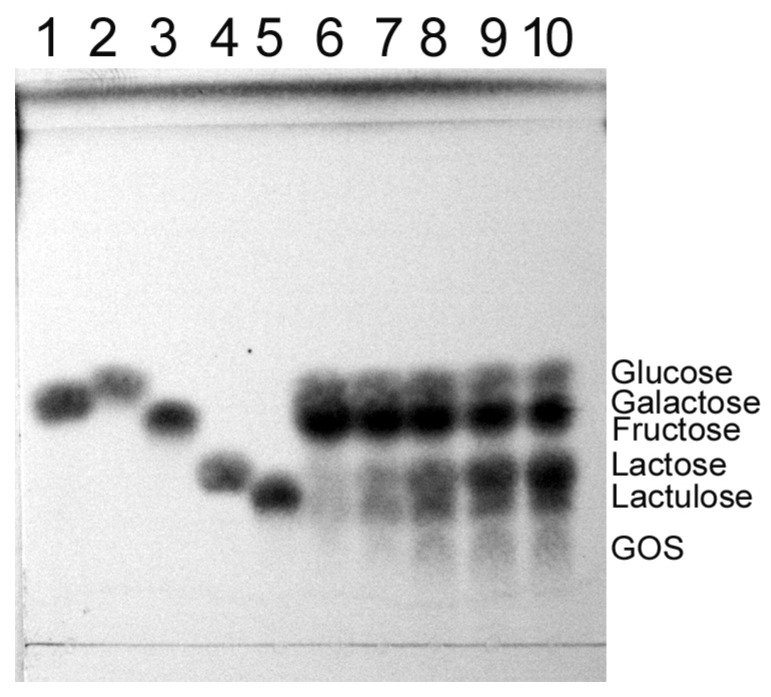
The TLC analysis of lactulose synthesis catalyzed by the *Arthrobacter* sp. S3* Bgal2 enzyme. Reaction mixtures containing 2 U mL^−1^ of *Arthrobacter* sp. S3* GH2 β-d-galactosidase and equimolar amounts of lactose and d-fructose as substrates were incubated at 15 °C for 8 h. Lane 1—d-galactose, lane 2—d-glucose, lane 3—d-fructose, lane 4—lactose, lane 5—lactulose, lane 6—29 mM concentration of each substrate in the reaction mixture, lane 7—58 mM concentration of each substrate in the reaction mixture, lane 8—146 mM concentration of each substrate in the reaction mixture, lane 9—292 mM concentration of each substrate in the reaction mixture, lane 10—438 mM concentration of each substrate in the reaction mixture.

**Figure 7:**
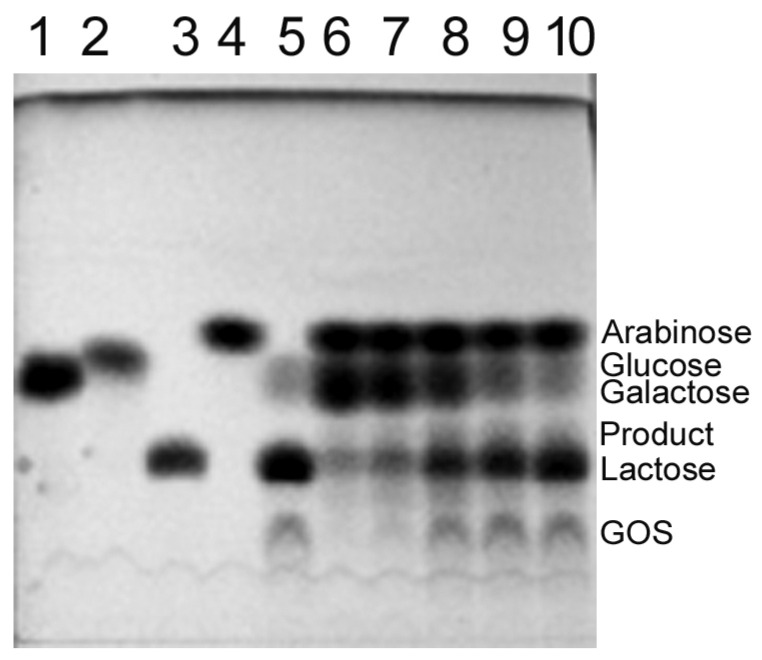
The TLC analysis of galactosyl-arabinose synthesis catalyzed by the *Arthrobacter* sp. S3* Bgal2 enzyme. Reaction mixtures containing 2 U mL^−1^ of *Arthrobacter* sp. S3* GH2 β-d-galactosidase and equimolar amounts of lactose and l-arabinose as substrates were incubated at 15 °C for 8 h. Lane 1—d-galactose, lane 2—d-glucose, lane 3—lactose, lane 4—l-arabinose, lane 5—reaction mixture after synthesis of GOS, lane 6—29 mM concentration of each substrate in the reaction mixture, lane 7—58 mM concentration of each substrate in the reaction mixture, lane 8—146 mM concentration of each substrate in the reaction mixture, lane 9—292 mM concentration of each substrate in the reaction mixture, lane 10—438 mM concentration of each substrate in the reaction mixture.

**Figure 8:**
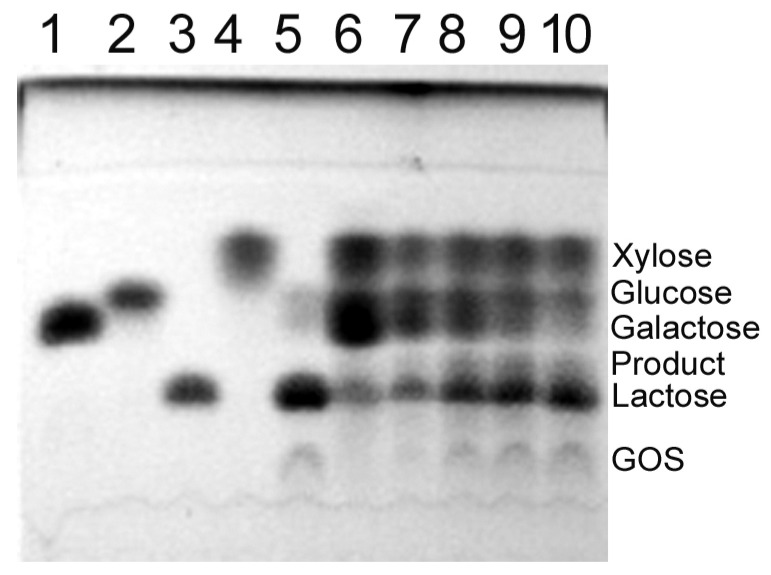
The TLC analysis of galactosyl-xylose synthesis catalyzed by the *Arthrobacter* sp. S3* Bgal2 enzyme. Reaction mixtures containing 2 U mL^−1^ of *Arthrobacter* sp. S3* GH2 β-d-galactosidase and equimolar amounts of lactose and d-xylose as substrates were incubated at 15 °C for 8 h. Lane 1—d-galactose, lane 2—d-glucose, lane 3—lactose, lane 4—d-xylose, lane 5—reaction mixture after synthesis of GOS, lane 6—29 mM concentration of each substrate in the reaction mixture, lane 7—58 mM concentration of each substrate in the reaction mixture, lane 8—146 mM concentration of each substrate in the reaction mixture, lane 9—292 mM concentration of each substrate in the reaction mixture, lane 10—438 mM concentration of each substrate in the reaction mixture.

The *Arthrobacter* sp. S3* β-d-galactosidases were also used to catalyze the synthesis of lactulose (galactosyl-fructose) from lactose and d-fructose as substrates. The Bgal42 was unable to synthesize lactulose (Figure S3A,B), but using Bgal2, significant amounts of lactulose were obtained in the reaction mixtures containing 58–292 mM of the substrates and 2 U mL^−1^ of the enzyme, after 8 h of incubation at 15 °C, with the optimum being at 146 mM (Figure 6). Similar results were achieved during the synthesis of other heterooligosaccharides, such as galactosyl-arabinose and galactosyl-xylose, from lactose and l-arabinose or d-xylose, respectively, when used as substrates (Figure 7 and Figure 8).

**Figure 9:**
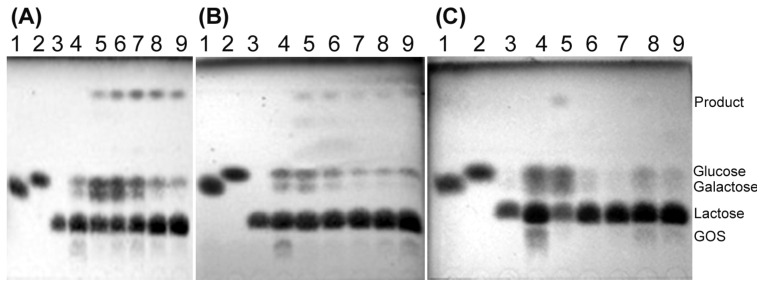
The TLC analysis of alkyl glycosides synthesis catalyzed by the *Arthrobacter* sp. S3* Bgal2 enzyme. Reaction mixtures containing 2 U mL^−1^ of *Arthrobacter* sp. S3* GH2 β-d-galactosidase and equimolar concentrations of lactose and 1-butanol (**A**), 1-hexanol (**B**) and 1-heptanol (**C**) as substrates were incubated at 15 °C for 8 h. Lane 1—d-galactose, lane 2—d-glucose, lane 3—lactose, lane 4—reaction mixture after synthesis of GOS, lane 5—29 mM concentration of each substrate in the reaction mixture, lane 6—58 mM concentration of each substrate in the reaction mixture, lane 7—146 mM concentration of each substrate in the reaction mixture, lane 8—292 mM concentration of each substrate in the reaction mixture, lane 9—438 mM concentration of each substrate in the reaction mixture.

Moreover, the *Arthrobacter* sp. S3* GH2 β-d-galactosidase catalyzed the synthesis of alkyl galactopyranosides from lactose and aliphatic alcohols such as 1-butanol, 1-hexanol and 1-heptanol at 15 °C. The enzyme exhibited the highest transglycosylation activity against 1-butanol, as the butyl galactopyranoside was detected in all tested reaction mixtures containing from 29 to 438 mM substrate concentrations, with the optimum being at 146 mM (Figure 9A). The yields of hexyl galactopyranoside were lower, probably due to the relatively low solubility of 1-hexanol in water (Figure 9B), and the heptyl galactopyranoside was detected only in the reaction mixture containing 29 mM concentrations of lactose and 1-heptanol (Figure 9C). In the case of 1-octanol, neither lactose hydrolysis nor transglycosylation products were detected, even at the lowest substrate concentrations of 29 mM, probably due to enzyme inactivation.

**Figure 10:**
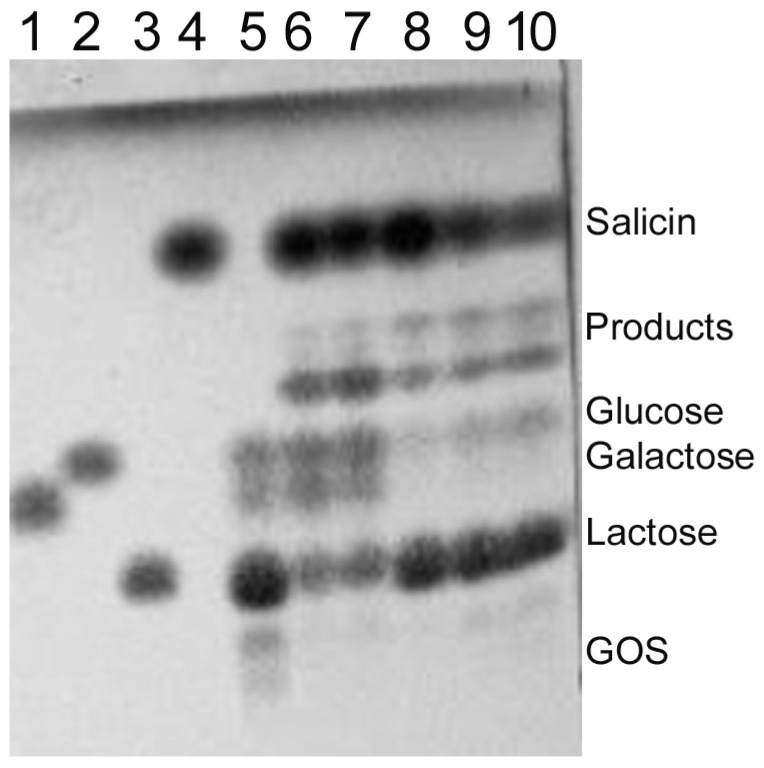
The TLC analysis of salicin glycosylation catalyzed by the *Arthrobacter* sp. S3* Bgal2 enzyme. Reaction mixtures containing 2 U mL^−1^ of *Arthrobacter* sp. S3* GH2 β-d-galactosidase and equimolar amounts of lactose and salicin as substrates were incubated at 15 °C for 9 h. Lane 1—d-galactose, lane 2—d-glucose, lane 3—lactose, lane 4—salicin, lane 5—reaction mixture after synthesis of GOS, lane 6—29 mM concentration of each substrate in the reaction mixture, lane 7—58 mM concentration of each substrate in the reaction mixture, lane 8—146 mM concentration of each substrate in the reaction mixture, lane 9—292 mM concentration of each substrate in the reaction mixture, lane 10—438 mM concentration of each substrate in the reaction mixture.

The transglycosylation of salicin (salicyl alcohol glucoside) was also performed using the *Arthrobacter* sp. S3* Bgal2 enzyme. In the reaction mixtures with low concentrations of both substrates (29 and 58 mM), one transglycosylation product dominated after 9 h of incubation at 15 °C, whereas at higher concentrations of lactose and salicin (146–438 mM), two products were efficiently synthesized (Figure 10).

## 3. Discussion

Bacteria belonging to the *Arthrobacter* genus are widely distributed in nature and have been isolated from various environments and various sources such as soil, water, air, plants and food (e.g., dairy products). Most *Arthrobacter* spp. produce pigments in a wide range of colors: yellow, orange, red, and even green and blue; this is often as an adaptation to extreme environments [33,34]. Several members of the genus *Arthrobacter* have been isolated from cold environments and are able to grow at low temperatures, so they have been classified as psychrophiles or psychrotolerants. For example, the *A. alpinus* strain S6-3, which was isolated from an alpine soil sampled at an altitude of 2300 m above sea level, grows at temperatures ranging from 1 to 25 °C and produces a yellow pigment. It also exhibits β-d-galactosidase activity and the ability to assimilate lactose [35]. In general, most of the described *Arthrobacter* sp. strains producing cold-active β-d-galactosidases have been isolated from soil samples, most frequently from Antarctic soil, namely *Arthrobacter* sp. strains C2-2, SB, 20B, ON14, 32c and 32cB [36,37,38,39,40,41,42].

According to the Carbohydrate Active Enzymes (CAZy) database (http://www.cazy.org/) [43], which describes families of structurally related catalytic domains and the carbohydrate-binding modules of enzymes that degrade, modify, or form glycosidic bonds, β-d-galactosidases mostly belong to GH1, GH2, GH35, and GH42. Their common features are the presence of a TIM-barrel type catalytic domain and a retaining mechanism of catalysis [3]. Some microorganisms, including bacteria of the *Arthrobacter* genus, produce several β-d-galactosidases belonging to different glycoside hydrolase families; for example, a psychrotolerant *Arthrobacter* sp. B7 isolated from Pennsylvania farmlands produces three isozymes belonging to GH2, GH35 and GH42 [44,45,46,47]. Therefore, for the efficient production and correct characterization of the enzyme, each β-d-galactosidase-encoding gene should be cloned and expressed in a heterologous host, e.g., the *E. coli* bacterium or *K. phaffii* yeast.

This article has described the identification, cloning, heterologous expression, purification and characterization of two β-d-galactosidases belonging to the GH2 and GH42 from the psychrotolerant Arctic bacterium *Arthrobacter* sp. S3*. It is interesting that the nucleotide sequences of the *bgal2* and *bgal42* genes and the amino acid sequences of the Bgal2 and Bgal42 enzymes are almost identical to the sequences of the *galA* and *galB* genes encoding GalA and GalB β-d-galactosidases from Antarctic *Arthrobacter* sp. ON14. Unfortunately, only the GalA enzyme has been partially characterized [40].

The multiple sequence alignment of *Arthrobacter* sp. S3* GH2 β-d-galactodidase (S3-Bgal2), with its counterparts from psychrotolerant *Arthrobacter* sp. C2-2 (C2-2-Bgal) and *Arthrobacter* sp. 32cB (32cB-Bgal), revealed some conserved regions and two essential catalytic residues, namely E448 and E527, corresponding to the E442 and E521 of *Arthrobacter* sp. C2-2 β-d-galactosidase (C2-2-1 isoenzyme) [48] and the E441 and E517 of *Arthrobacter* sp. 32cB β-d-galactosidase (Figure S1) [30,49]. The Trp548 residue, which is located in the bottom of the 32cB-Bgal active site [30], corresponds to W552 of the C2-2-Bgal and W558 of the S3-Bgal2 (Figure S1). In all three enzymes, Asp, His and Asn residues directly involved in binding the galactosyl moiety of the substrate (e.g., lactose) are also present, namely D207, H368, N440 and H520 of the *Arthrobacter* sp. 32cB β-d-galactosidase [30]; D201, H369, N441 and H524 of the *Arthrobacter* sp. C2-2 β-d-galactosidase; and D201, H375, N447 and H530 of the *Arthrobacter* sp. S3* GH2 β-d-galactosidase (Figure S1). In the case of 32cB-Bgal, the rotation of the side chain of phenylalanine 581 plays a key role in transferring the substrate from the shallow to the deep binding mode. In GH2 family β-d-galactosidases from *Arthrobacter* sp. C2-2 and *Arthrobacter* sp. S3*, these are F585 and F591 residues, respectively. Furthermore, the Trp999 residue in *E. coli* LacZ, responsible for stabilizing the glucosyl moiety of lactose [50], was replaced by a Cys residue in all three cold-active enzymes, namely C985 in 32cB-Bgal, C999 in C2-2-Bgal and C1005 in S3-Bgal2 (Figure S1), which may affect the ability to transfer the galactosyl moiety to different acceptors during transglycosylation [30,48]. Another amino acid residue, Asn110, which probably plays a role in the synthesis of heterooligosaccharide lactulose catalyzed by 32cB-Bgal, was also identified [49], and this corresponds to N100 residues in both C2-2-Bgal and S3-Bgal2 (Figure S1). All this indicates the high conservation of amino acid residues involved in the hydrolytic and transglycosylation activity of β-d-galactosidases derived from psychrotolerant bacteria of the *Arthrobacter* genus and may help in their rational improvement by site-directed mutagenesis.

The *Arthrobacter* sp. S3* Bgal2 exists as a tetramer in its native form, like many other cold-active GH2 β-d-galactosidases characterized to date (Table 6) and *E. coli* LacZ [50]; meanwhile, the *Arthrobacter* sp. 32cB β-d-galactosidase is a dimer and the enzyme from *Arthrobacter* sp. C2-2 is a hexamer (Table 6). The main industrial β-d-galactosidase from *Kluyveromyces lactis* yeast is active in both dimeric and tetrameric forms [51].

The recombinant *Arthrobacter* sp. S3* Bgal2 enzyme can be efficiently produced in *E. coli* cells by reducing the temperature of cultivation from 37 to 30 °C. The expression of the *bgal2* gene at 37 °C was much lower, probably due to the instability of the recombinant Bgal2 protein, whereas at 25 °C, the growth of *E. coli* was poor, resulting in reduced enzyme production. Unfortunately, the molecular weight of native β-d-galactosidase is too large for the efficient secretion of the protein by *K. phaffii* yeast cells. A similar result was obtained previously for the tetrameric cold-active β-d-galactosidase from *Pseudoalteromonas* sp. 22b [41].

The *Arthrobacter* sp. S3* Bgal2 enzyme is cold-active, with maximum activity at 22 °C under the conditions tested (20 mM of potassium phosphate buffer with 50 mM of KCl, pH 7.5). Only a few β-d-galactosidases isolated from cold-adapted microorganisms are optimally active at temperatures below 20 °C (Table 6 and Table 7), and only one enzyme, which was isolated from *A. psychrolactophilus* F2, shows maximum activity at 10 °C [52,53]. The GalA β-d-galactosidase from *Arthrobacter* sp. ON14, the enzyme with the highest sequence similarity to the *Arthrobacter* sp. S3* Bgal2, exhibited maximum activity at 15 °C [40]. The Bgal2 from *Arthrobacter* sp. S3* can be easily inactivated by incubation for a few minutes at a moderate temperature (5 min at 45 °C), which is important for industrial enzymes because it allows for the easy management of the process. The *Arthrobacter* sp. ON14 GalA was quite stable at 45 °C, and it was inactivated by incubation at 50 °C for 20 min [40].

The slightly alkaline optimum pH of the *Arthrobacter* sp. S3* Bgal2 is within the range reported for other bacterial GH family 2 β-d-galactosidases (Table 6), whereas at pH 6.5 (a value close to the pH of milk), the enzyme showed half of its maximum activity.

The Bgal2 enzyme from *Arthrobacter* sp. S3* exhibits high specificity towards β-d-galactopyranosides, similar to other cold-active β-d-galactosidases belonging to GH family 2. It efficiently hydrolyzes the synthetic substrates ONPG and PNPG, and the natural substrate lactose, whereas the recombinant *Paracoccus* sp. 32d β-d-galactosidase exhibits relatively high activity towards *p*NP-β-d-fucopyranoside; however, this enzyme does not have the C-terminal Bgal_small_N domain [54]. β-d-Galactosidases from *Alkalilactibacillus ikkense* and *Arthrobacter* sp. 32cB also show weak activity towards *p*NP-β-d-fucopyranoside [42,55]. The enzyme from *Arthrobacter* sp. 20B is able to digest *p*NP-β-d-mannopyranoside [39], and the *Pseudoalteromonas* sp. 22b β-d-galactosidase shows very weak activity towards *p*NP-β-d-galacturonide [56,57].

A comparison of the activity of cold-active GH2 β-d-galactosidases against the natural substrate revealed that at 10 °C, the *Arthrobacter* sp. S3* Bgal2 exhibits higher catalytic efficiency towards lactose than enzymes from *A. psychrolactophilus* F2 [52,53], *Arthrobacter* sp. SB [38] and *Arthrobacter* sp. C2-2 [37], and a similar catalytic activity (*k*_cat_/*K*_m_) towards β-d-galactosidases from *Arthrobacter* sp. 32cB [42] and *Alteromonas* sp. ML117 [58]. The kinetic parameters of the GalA enzyme from *Arthrobacter* sp. ON14 were not determined.

**Table 6:** Biochemical properties of cold-active GH2 β-d-galactosidases from various sources.

Source	Molecular Mass (kDa)	Oligomeric State	T_opt_ (°C)	pH_opt_	Thermal Inactivation	Kinetic Parameters of Lactose Hydrolysis			References
						*K*_m_ (mM)	*k*_cat_ (s^−1^)	*k*_cat_/*K*_m_ (s^−1^ mM^−1^)	
*Arthrobacter* sp. S3*	450 (111.6) ^a^	4	22	7.5	5 min at 45 °C	11.70 (10 °C)	20.50	1.75	This study
*Arthrobacter psychrolactophilus* F2	548 (111.7) ^a^	4	10	8.0	5 min at 50 °C	50 ^b^, 42.1 ^c^ (10 °C)	18.0 ^b^, 12.7 ^c^	0.36 ^b^, 0.30 ^c^	[52,53]
*Arthrobacter* sp. ON14	NR (111.4) ^a^	NR	15	8.0	20 min at 50 °C	NR	NR	NR	[40]
*Arthrobacter* sp. SB	463 (114.0) ^a^	4	18	7.0	10 min at 37 °C	11.5 (20 °C)	5.2	0.53	[38]
*Arthrobacter* sp. 20B	460 (113.7) ^a^	4	25	6.0–8.0	1 min at 60 °C	NR	NR	NR	[39]
*Arthrobacter* sp. 32cB	257 (109.6) ^a^	2	28	8.0	5 min at 44 °C	16.56 (10 °C)	31.84	1.92	[42]
*Arthrobacter* sp. B7	NR (111.0) ^a^	NR	40	7.2	10 min at 50 °C	16 (30 °C)	NR	NR	[45]
*Arthrobacter* sp. C2-2	660 (110.8) ^a^	6	40	7.5	10 min at 50 °C	344.2 (10 °C)	324	0.9	[37,48]
*Alkalilactibacillus ikkense*	NR (119.1) ^a^	NR	20–30	8.0	5 min at 50 °C	NR	NR	NR	[55]
*Alteromonas* sp. ML117	450 (117.9) ^a^	4	30	8.0	10 min at 35 °C	3.8 (10 °C)	7.8	2.1	[58]
*Pseudoalteromonas* sp. 22b	490 (117.1) ^a^	4	40	6.0–8.0	2 min at 50 °C	3.3 (20 °C)	157	47.5	[56,57]

NR not reported; ^a^ molecular weight of the monomer, calculated from the amino acid sequence; ^b^ protein isolated from natural host; ^c^ recombinant protein.

It was found that neither d-glucose nor d-galactose had any effect on the activity of *Arthrobacter* sp. S3* Bgal2. A similar behavior was reported for the *Arthrobacter* sp. C2-2 β-d-galactosidase [37]. The cold-active GH2 β-d-galactosidases originating from *Arthrobacter* sp. SB and *Arthrobacter* sp. 32cB are inhibited by d-galactose [38,42], which is the competitive inhibitor of many microbial β-d-galactosidases, including a commercial enzyme from *Kluyveromyces fragilis* (Lactozym, Novo Nordisk, Denmark) [59,60]. The β-d-galactosidase from *Pseudoalteromonas* sp. 22b is inhibited by d-glucose, but not by d-galactose [61], whereas the enzyme from *Paracoccus* sp. 32d was inhibited by both monosaccharides [54]. In addition, the calcium present in milk can also be an inhibitor of β-d-galactosidases. A 5 mM concentration of Ca^2+^ in the reaction mixture reduces the activity of the *Arthrobacter* sp. S3* Bgal2 enzyme, as well as β-d-galactosidases from *Arthrobacter* sp. 32cB, *Paracoccus* sp. 32d and *Alteromonas* sp. ML117 [42,54,58]; however, it has little effect on the glycoside hydrolase family 2 from *Arthrobacter* sp. 20B and *Arthrobacter* sp. ON14 [39,40].

Despite this, *Arthrobacter* sp. S3* Bgal2 could be used in the dairy industry for the removal of lactose from milk under refrigeration. One unit of this enzyme is able to hydrolyze 85% of the lactose in 1 mL of milk in 24 h at 10 °C. This can be explained by the fact that milk is a multi-component matrix containing protein, fat, lactose and minerals such as calcium, magnesium, potassium and sodium, as well as inorganic phosphate, chloride and citrate, which are associated with each other and with proteins, mainly casein micelles. According to A. Tsioulpas et al., the average concentration of free Ca^2+^ ions (β-d-galactosidase inhibitor) in cows’ milk is 1.88 mM and ranges from 1.05 to 5.29 mM, depending on the sample [62]. On the other hand, the concentration of free Mg^2+^ ions (enzyme activator) is estimated to be 0.81 mM [63]. Ultimately, the efficiency of lactose hydrolysis in milk depends on the influence of both divalent cations on the β-d-galactosidase enzyme, as well as monovalent Na^+^ and K^+^. In our study, potassium ions stabilized the structure of the *Arthrobacter* sp. S3* Bgal2 enzyme. For comparison, 1 U of *Arthrobacter* sp. 32cB β-d-galactosidase digests 70%, 1 U of *A. psychrolactophilus* F2 enzyme hydrolyzes about 80%, 1 U of *Alteromonas* sp. ML117 digests 86%, while 1 U of *Paracoccus* sp. 32d β-d-galactosidase removes approximately 97% of lactose from 1 mL of milk under the same conditions [42,52,54,58]. After incubation with 200 µg (about 5 U) of purified GalA enzyme from *Arthrobacter* sp. ON14 in 1 mL of milk at 4 °C for 8 h, the lactose was completely digested into d-glucose and d-galactose [40].

This article also presents studies on a second β-d-galactosidase from *Arthrobacter* sp. S3*, a member of the GH family 42. The multiple sequence alignment of *Arthrobacter* sp. S3* Bgal42 (S3-Bgal42), with its counterparts from the psychrotolerant bacterium *Rahnella* sp. R3 (RR3-Bgal) and thermophilic *Thermus thermophilus* A4 (TT-Bgal), revealed two essential catalytic residues, namely E172 and E330, corresponding to E157 (acid/base catalyst) and E314 (nucleophile) of the *Rahnella* sp. R3 β-d-galactosidase, and E141 and E312 of the *T. thermophilus* A4 β-d-galactosidase (Figure S2) [31,32]. The conserved catalytic residues E183 and E341 are also present in the amino acid sequence of GH family 42 β-d-galactosidase from psychrotolerant *Arthrobacter* sp. 32c [41], which shows very high similarity to the S3-Bgal42 sequence (Table 2, Figure S2). In addition, eight highly conserved residues putatively involved in d-galactose binding were found in all four amino acid sequences, namely R118, N156, W194, D276, W322, F352, E362 and H365 of the RR3-Bgal [32]; R102, N140, W182, D264, W320, F350, E360 and H363 of the TT-Bgal; R133, N171, W212, D296, W338, F368, E378 and H381 of the S3-Bgal42; and R144, N182, W223, D307, W349, F379, E389 and H392 of 32c-Bgal (Figure S2). Importantly, both β-d-galactosidases from *Arthrobacter* sp. do not possess the metal ion-binding site composed of four cysteine residues present in other enzymes belonging to GH family 42, i.e., C122, C162, C164 and C167 of the *Rahnella* sp. R3 β-d-galactosidase; C106, C150, C152 and C155 of the *T. thermpphilus* A4 enzyme (Figure S2) [31,32]; or C115, C155, C157 and C160 of the *Bacillus circulans* sp. *alkalophilus* β-d-galactosidase [64]. In the amino acid sequences of S3-Bgal42 and 32c-Bgal, only two Cys residues have been found, namely C175, C480 and C186, and C491, respectively (Figure S2).

The *Arthrobacter* sp. S3* Bgal42 exists as a trimer in its native form; this is in addition to β-d-galactosidases from psychrotolerant *Arthrobacter* sp. 32c, *Rahnella* sp. R3 and thermophilic *T. thermophilus* A4 [31,41,65]. The cold-active GH family 42 β-d-galactosidases from the *Planococcus* sp. strains L4 and SOS Orange are dimers [66,67], whereas the *Marinomonas* sp. ef1 enzyme, which combines cold activity with a high optimal temperature and thermostability, forms a hexamer in solution and in the crystal structure [68].

The relatively low molecular weight of the *Arthrobacter* sp. S3* Bgal42 protein enables its efficient heterologous production in *E. coli* cells and secretion by *K. phaffii* cells, although the production of the enzyme in yeast requires optimization. The GH42 β-d-galactosidases from *Arthrobacter* sp. 32c, *Planococcus* sp. L4 and a metagenome-derived ZD410 enzyme were also produced in the *K. phaffii* expression system and efficiently secreted into the culture medium [41,69,70].

The optimal pH and temperature of the *Arthrobacter* sp. S3* Bgal42 activity are within the ranges reported for other GH family 42 β-d-galactosidases (Table 7). It is a cold-adapted enzyme because it exhibits relatively high activity, around 40%, at low temperatures from 5 to 10 °C, and it can be easily inactivated by a 10 min incubation at a moderate temperature of 50 °C. Meanwhile, the *E. coli* crude extract containing the recombinant GalB enzyme from *Arthrobacter* sp. ON14, the closest homologue of the *Arthrobacter* sp. S3* Bgal42, showed maximum activity at 37 °C, and had a very low activity at 10 °C [40]. Only one β-d-galactosidase belonging to GH family 42, originating from *Planococcus* sp. L4, has much better properties. This enzyme shows maximum activity at 20 °C, around 80% of maximum activity at 10 °C, and as much as 27% of maximum activity at 0 °C [66].

**Table 7:** Biochemical properties of cold-active GH42 β-d-galactosidases from various sources.

Source	Molecular Mass (kDa)	Oligomeric State	T_opt_ (°C)	pH_opt_	Thermal Inactivation	Kinetic Parameters of Lactose Hydrolysis			References
						*K*_m_ (mM)	*k*_cat_ (s^−1^)	*k*_cat_/*K*_m_ (s^−1^ mM^−1^)	
*Arthrobacter* sp. S3*	233 (74.4) ^a^	3	40	7.0	10 min at 50 °C	1.17 (10 °C)	0.34	0.29	This study
*Arthrobacter* sp. 32c	195 (75.9) ^a^	3	50	6.5	NR	77.54 (10 °C)	1.76	0.023	[41]
*Arthrobacter* sp. B7	NR (70.9) ^a^	NR	45–50	6.6	15 min at 50 °C	4.81 (30 °C)	NR	NR	[47]
*Planococcus* sp. L4	156 (77.3) ^a^	2	20	6.8	10 min at 45 °C	11.2 (10 °C)	62.0	5.5	[66]
*Planococcus* sp. SOS orange	155 (77.5) ^a^	2	42	6.5	10 min at 55 °C	NR	NR	NR	[67]
*Cryobacterium* sp. LW097 (Bgal322)	NR (74.8) ^a^	NR	25	6.0	(10 min at 55 °C) ^b^	18.1 (5° C)	54.0	3.0	[71]
*Cryobacterium* sp. LW097 (Bgal435)	NR (77.6) ^a^	NR	30	6.5	(10 min at 55 °C) ^b^	28.2 (5 °C)	68.2	2.4	[71]
*Cryobacterium* sp. LW097 (Bgal2567)	NR (74.3) ^a^	NR	35	6.5	(10 min at 55 °C) ^b^	13.5 (5 °C)	22.2	1.7	[71]
*Carnobacterium maltaromaticum* BA	NR (76.8) ^a^	NR	30	NR	10 min at 40 °C	NR	NR	NR	[72]
*Rahnella* sp. R3	225 (77.1) ^a^	3	35	6.5	5 min at 55 °C	2.2 (4 °C)	2.5	1.1	[32,65]
Soil metagenome	NR (78.6) ^a^	NR	38	7.0	60 min at 50 °C	27.1 (10 °C)	33.0	1.21	[70]
*Marinomonas* sp. ef1	396 (74.3) ^a^	6	55	6.0	7 days at 50 °C	NR	NR	NR	[68]

NR not reported; ^a^ molecular weight of the monomer, calculated from the amino acid sequence; ^b^ less than 10% of initial activity.

The *Arthrobacter* sp. S3* Bgal42 shows high substrate specificity, with high activity towards chromogenic β-d-galactopyranosides (ONPG, PNPG) and low activity towards *p*NP-β-d-fucopyranoside. Similar behavior was reported for the β-d-galactosidase from *Planococcus* sp. SOS Orange [67]. The enzyme from *Arthrobacter* sp. 32c displayed high activity towards chromogenic β-d-galactopyranosides, and slight activity towards chromogenic β-d-glucopyranoside [41]. The Bgal435 from *Cryobacterium* sp. LW097 also had β-glucosidase activity [71]. The β-d-galactosidase from *Planococcus* sp. L4 hydrolyzed chromogenic β-d-galactopyranosides only [66], whereas the glycoside hydrolase family 42 from the lactic acid bacterium *Carnobacterium maltaromaticum* BA (formerly *C. piscicola* BA) was active towards ONPG, PNPG, *p*NP-β-d-fucopyranoside and *p*NP-β-d-glucuronide [72]; in addition, the metagenome-derived ZD410 enzyme efficiently hydrolyzed chromogenic β-d-galactopyranosides, *p*NP-β-d-mannoside, *p*NP-β-d-arabinoside and *p*NP-β-d-glucuronide [70].

The GH family 42 β-d-galactosidase from *Arthrobacter* sp. S3* is also able to hydrolyze natural β-d-galactopyranoside lactose. At 10 °C, the enzyme exhibits almost 13 times higher catalytic efficiency (*k*_cat_/*K*_m_) towards lactose than β-d-galactosidase from *Arthrobacter* sp. 32c (Table 7). Unfortunately, other β-d-galactosidases derived from the cold-adapted bacteria *Planococcus* sp. L4, *Rahnella* sp. R3 and *Cryobacterium* sp. LW097, as well as the metagenome-derived ZD410 enzyme, digested lactose much more efficiently than *Arthrobacter* sp. S3* Bgal42 (Table 7).

On the other hand, the GH42 β-d-galactosidase from *Arthrobacter* sp. S3* is strongly activated by calcium ions, which is very advantageous in the processing of milk. The GH42 β-d-galactosidase from *Rahnella* sp. R3 and the ZD410 enzyme were only slightly stimulated by Ca^2+^ [65,70]. Calcium ions had no effect on the activity of β-d-galactosidases from *Planococcus* sp. L4 and *Planococcus* sp. SOS Orange [66,67], and inhibited enzymes from *Arthrobacter* sp. 32c and *Cryobacterium* sp. LW097 [41,71].

The *Arthrobacter* sp. S3* Bgal42 exhibits relatively low lactose hydrolysis in milk. After 2 h of incubation at 10 °C, only 1.4% of lactose was hydrolyzed by 1 U (8.7 μg)/mL of the enzyme. For comparison, after 60 min of incubation at 4 °C, 4.2% of disaccharide was digested by 1 U/mL of the metagenome-derived ZD410 β-d-galactosidase [70], and 36% of milk lactose was even hydrolyzed by 2.5 µg of the *Planococcus* sp. L4 β-d-galactosidase after 60 min at 5 °C [66]. The low efficiency of lactose hydrolysis is probably caused by the inhibition of the *Arthrobacter* sp. S3* Bgal42 by both d-glucose and d-galactose. The *Planococcus* sp. L4 β-d-galactosidase and two enzymes from *Cryobacterium* sp. LW097 (Bgal435 and Bgal2567) were also inhibited by d-galactose [66,71], while d-glucose was a strong inhibitor of the *Arthrobacter* sp. 32c β-d-galactosidase [41].

In our studies, we tested not only the hydrolytic but also the transglycosylation activity of both enzymes originating from the Arctic bacterium *Arthrobacter* sp. S3*. Both β-d-galactosidases were capable of synthesizing GOS (tri- and tetrasaccharides) at relatively low temperatures ranging from 10 to 20 °C for the Bgal2, and at 30 °C for the Bgal42. The formation of GOS during lactose hydrolysis has previously been noted for cold-active β-d-galactosidases belonging to GH family 2 from *A. psychrolactophilus* F2 [53], *Arthrobacter* sp. C2-2 [37], *Arthrobacter* sp. 32cB [42] and *Alkalilactibacillus ikkense* [55]. In the case of recombinant *Arthrobacter* sp. C2-2 β-d-galactosidase, the tri- and tetrasaccharides were formed at 15 °C [37]. The product corresponding to trisaccharide was detected during lactose hydrolysis in milk catalyzed by *A. psychrolactophilus* F2 β-d-galactosidase, at 10 °C [53]. The *Arthrobacter* sp. 32cB β-d-galactosidase synthesized tri-, tetra- and pentasaccharides at temperatures ranging from 10 to 30 °C [42].

Moreover, it was found that the *Arthrobacter* sp. S3* Bgal2 catalyzed the synthesis of heterooligosaccharides such as lactulose (galactosyl-fructose), galactosyl-arabinose, galactosyl-xylose and glycosylated salicin at 15 °C, using the natural, plentiful and inexpensive substrate lactose as a galactosyl donor. Similar results were obtained for the *Arthrobacter* sp. 32cB β-d-galactosidase, but at a higher temperature of 30 °C [42]. Unfortunately, it was not possible to synthesize lactulose using the *Arthrobacter* sp. S3* Bgal42 enzyme (Figure S3A,B). The lack of detectable amounts of product could be a result of the enzyme’s inability to transfer the galactosyl moiety to the fructose acceptor. The resulting heterodisaccharide could also be preferentially hydrolyzed. According to L. Wang et al., the Bgal322 and Bgal2567 enzymes belonging to the GH family 42 from the *Cryobacterium* sp. LW097 hydrolyzed lactulose more than twice as efficiently as lactose [71]; therefore, we decided to investigate the preferences of the *Arthrobacter* sp. S3* Bgal42 enzyme towards lactose and lactulose. The hydrolysis of both substrates at a concentration of 120 mM was carried out for 24 h at 10 °C with 35 U mL^−1^ of the β-d-galactosidase, and then the reaction products were analyzed by HPLC. The efficiency of lactose hydrolysis was 58%, while lactulose was almost completely digested (Figure S4A,B). The hydrolysis of lactulose catalyzed by the *Arthrobacter* sp. S3* Bgal2 enzyme (8 U mL^−1^) was also performed under the same conditions. In this case, 51% substrate loss was obtained and a significant number of oligosaccharides were formed (Figure S5). The obtained results confirmed the much better transglycosylation activity of the *Arthrobacter* sp. S3*β-d-galactosidase from GH family 2 compared to the enzyme from GH42. In the next study, we plan to test the suitability of *Arthrobacter* sp. S3* Bgal2 for the synthesis of heterotrisaccharide lactosucrose.

The *Arthrobacter* sp. S3* GH2 β-D-galactosidase was also investigated as a tool for the synthesis of alkyl galactopyranosides. It catalyzed the synthesis of 1-butyl and 1-hexyl galactopyranosides, like cold-active β-d-galactosidases from the *Arthrobacter* sp. 32cB [42] and the *Pseudoalteromonas* sp. 22b [73], but at a lower temperature of 15 °C. The Bgal2 enzyme was also capable of catalyzing the formation of 1-heptyl galactopyranoside, but with low efficiency. A decrease in the efficiency of alkyl galactopyranosides synthesis with an increase in the length of the hydrocarbon chain of the primary alcohol was also noted for the *Pseudoalteromonas* sp. 22b β-d-galactosidase [73]. However, the efficiency of alkyl galactopyranosides synthesis can be increased by the addition of selected organic solvents, reducing the water activity in the reaction mixture, and via the optimization of the reaction conditions, which we plan to investigate.

## 4. Materials and Methods

### 4.1. Chemical Reagents and Ingredients of Culture Media

5-Bromo-4-chloro-3-indolyl-β-d-galactopyranoside (X-gal) and isopropyl-β-d-thiogalactopyranoside (IPTG) were purchased from Biosynth AG (Staad, Switzerland). Tributyrine, starch (soluble), Tween 80, dithiothreitol (DTT), tris(2-carboxyethyl)phosphine (TCEP), ethylenediaminetetraacetic acid sodium salt (EDTA), N-(2-hydroxyethyl)piperazine-N’-(2-ethanesulfonic acid) (HEPES), 1-butanol, 1-hexanol, 1-heptanol, 1-octanol, *o*-nitrophenyl-β-d-galactopyranoside (ONPG), *p*-nitrophenyl-β-d-galactopyranoside (PNPG), *p*-nitrophenyl-α-d-galactopyranoside, *p*-nitrophenyl-β-d-glucopyranoside, *p-*nitrophenyl-α-d-glucopyranoside, *p-*nitrophenyl-β-d-glucuronide, *p-*nitrophenyl-β-d-fucopyranoside, *p-*nitrophenyl-β-l-arabinopyranoside, *p-*nitrophenyl-β-d-xylopyranoside, *p-*nitrophenyl-β-d-mannopyranoside, *p-*nitrophenyl-β-d-cellobioside, l-arabinose, d-xylose, lactulose (4-O-β-d-galactopyranosyl-d-fructose), biotin and Yeast Nitrogen Base Without Amino Acids were supplied by Sigma (St. Louis, MO, USA). Salicin (2-(hydroxymethyl)phenyl-β-d-glucopyranoside) was obtained from AppliChem GmbH (Darmstadt, Germany). Marine salt was purchased from Dohse Aquaristik GmbH & Co. KG (Grafschaft, Germany). Peptone K, yeast extract and bacteriological agar were supplied by BTL (Łódź, Poland). All other chemicals were supplied by POCH (Gliwice, Poland).

### 4.2. Isolation, Characterization and Identification of Bacterial Strain with β-d-Galactosidase Activity

First, 0.5 g of Arctic soil was dissolved in 10 mL of sodium chloride physiological solution and, after decantation, 250 μL aliquots of the supernatant were spread out on LAS agar plates (1% marine salt, 0.5% peptone K, 0.25% yeast extract, 1.5% bacteriological agar) and incubated at 20 °C for 72–96 h. The proteolytic, esterolytic, lipolytic, amylolytic and β-d-galactosidase activities of the obtained bacterial strains were examined at 24 °C on agar plates containing 25% skimmed milk, 1% tributyrine, 1% Tween 80 and 0.01% CaCl_2_, 2% starch, and X-gal and IPTG (20 and 24 μg mL^−1^), respectively. The growth properties of the analyzed S3* isolate were determined in LBS medium (1% marine salt, 0.5% peptone K, 0.25% yeast extract, pH 7.0). Gram staining was performed using a Gram-color stain set for the Gram staining method (Merck KGaA, Darmstadt, Germany).

Bacterial chromosomal DNA was isolated using Genomic Mini AX Bacteria (A&A Biotechnology, Gdynia, Poland), and it was used as a template to amplify the 16S rDNA gene with fD1 and rP2 primers [74]. The PCR product was purified from an agarose gel band, cloned into a pJET1.2/blunt vector using the CloneJet^TM^ PCR Cloning Kit (Thermo Fisher Scientific Baltics, Vilnius, Lithuania), and sequenced (Genomed, Warsaw, Poland).

### 4.3. Preparation of the Arthrobacter sp. S3* Genomic Library

The genomic DNA of *Arthrobacter* sp. S3* was digested with *Bgl*II and *Hin*dIII restriction endonucleases (Thermo Fisher Scientific Baltics, Vilnius, Lithuania) at 37 °C for 2 h, and purified by a sodium acetate/ethanol precipitation. Chromosomal DNA fragments were cloned into a pBAD/*Myc*-His A vector (Invitrogen, Carlsbad, CA, USA), which was digested with the same enzymes. The ligated DNA was transformed into *E. coli* TOP10F’ chemically competent cells (Invitrogen, Carlsbad, CA, USA), and the resulting DNA library was screened on LB agar plates (1% NaCl, 1% peptone K, 0.5% yeast extract, 1.5% bacteriological agar) supplemented with ampicillin (100 µg mL^−1^), X-gal (20 μg mL^−1^) and IPTG (24 μg mL^−1^). The plates were incubated at 37 °C for 24 h and then transferred to 24 °C for 48 h. After incubation, the bacterial colonies producing β-d-galactosidase were blue in color. The library plasmids from positive transformants were isolated using the Plasmid Mini kit (A&A Biotechnology, Gdynia, Poland), and the *Arthrobacter* sp. S3* genomic DNA inserts were sequenced (Genomed, Warsaw, Poland).

### 4.4. Construction of E. coli Expression Systems for the Production of Arthrobacter sp. S3* β-d-Galactosidases

The *bgal2* gene was amplified by PCR using S3BgNNco 5′ ATAT**CCATGG**TGACCCCCGCAGACGTTTCGTACATCACC 3′ and S3BgCHind 5′ AGCG**AAGCTT****A**CAGTGCGGAGAAGCGCAGTACCAGCGT 3′ primers, with *Nco*I and *Hin*dIII recognition sites (underlined), respectively. The start and stop codons are bolded. The pBAD/β-galS3* Lib1 plasmid was used as a template. The PCR product of 3107 bp was purified and digested with the *Alw*NI restriction enzyme (New England BioLabs, Ipswich, MA, USA). The restriction fragments were separated by electrophoresis and purified from agarose gel bands using a DNA Gel-Out kit (A&A Biotechnology, Gdynia, Poland). The DNA fragment of 987 bp was then digested with *Nco*I endonuclease, whereas the DNA fragment of 2120 bp was digested with the *Hin*dIII restriction enzyme purchased from Thermo Fisher Scientific Baltics (Vilnius, Lithuania). At the same time, the pBAD/*Myc*-His A vector (Invitrogen, Carlsbad, CA, USA) was digested with *Nco*I and *Hin*dIII endonucleases. After purification, three DNA fragments were ligated using DNA ligase T4 (Epicentre Biotechnologies, Madison, WI, USA) and transformed into *E. coli* TOP10 chemically competent cells (Invitrogen, Carlsbad, CA, USA). The positive clones were selected on LB agar plates (1% NaCl, 1% peptone K, 0.5% yeast extract, 1.5% bacteriological agar) supplemented with ampicillin (100 µg mL^−1^), X-gal (20 μg mL^−1^) and l-arabinose (200 μg mL^−1^). The plates were incubated at 37 °C for 12 h and then transferred to 24 °C for another 12 h. After incubation, the bacterial colonies producing *Arthrobacter* sp. S3* GH2 β-d-galactosidase (Bgal2) were blue. The pBAD/Myc-HisA-GH2β-galS3* expression plasmid was isolated using the Plasmid Mini kit (A&A Biotechnology, Gdynia, Poland), and sequenced. The scheme of recombinant pBAD/*Myc*-HisA-GH2β-galS3* plasmid construction is shown in Figure S6.

The *bgal42* gene was amplified using forward primer BgalS3FNco 5′ TAT**CCATGG**CGTCCCCGATCCCCGAGAAATC 3′ and reverse primer BgalS3RHind 5′ GCG**AAGCTT****A**GGACTCGGCGATGACGGCTACGGC 3′ (containing *Nco*I and *Hin*dIII recognition sites, respectively). PCR was performed using DNA polymerase *Hypernova* (Blirt, Gdańsk, Poland) and the pBAD/β-galS3* Lib2 plasmid as a template. The PCR product of 2064 bp was then purified from the reaction mixture and digested with *Nco*I and *Pst*I endonucleases (Thermo Fisher Scientific Baltics, Vilnius, Lithuania). The restriction fragments were separated by electrophoresis. The DNA fragment of 1218 bp was purified from an agarose gel band using a DNA Gel-Out kit (A&A Biotechnology, Gdynia, Poland), and cloned into the pBAD/*Myc*-His A vector (Invitrogen, Carlsbad, CA, USA) digested with the same restriction enzymes. The obtained pBAD-N-endBgalS3* recombinant plasmid contained the *Nco*I-*Pst*I fragment of the *bgal42* gene. Afterwards, the PCR product was digested with *Pst*I and *Hin*dIII restriction enzymes. After electrophoresis, the DNA fragment of 834 bp was purified and cloned into the pBAD-N-endBgalS3* plasmid digested with the same endonucleases. The resulting pBAD-GH42BgalS3* recombinant plasmid contained the *Arthrobacter* sp. S3* *bgal42* gene under the control of the *ara*BAD (P_BAD_) promoter. The scheme of recombinant pBAD-GH42BgalS3* plasmid construction is shown in Figure S7.

### 4.5. Construction of K. phaffii Expression Systems for the Production of Arthrobacter sp. S3* β-d-Galactosidases

The *bgal2* gene encoding *Arthrobacter* sp. S3* GH2 β-d-galactosidase was amplified using two primers: FS3sklBgXho 5′ CTATG**CTCGAG**AAAAGA**ATGACCCCCGCAGACGTTTCGTACATCACC** 3′ and RS3sklBgXba 5′ GACTG**TCTAGA****TTACAGTGCGGAGAAGCGCAGTACCAGCGT** 3′, with *Xho*I and *Xba*I recognition sites (sequences complementary to the template are given in bold). The PCR was performed using DNA polymerase *LongNova* (Blirt, Gdańsk, Poland) and the pBAD/Myc-HisA-GH2β-galS3* recombinant plasmid as a template. The PCR product of 3118 bp was purified, digested with *Xho*I and *Xba*I restriction enzymes (Thermo Fisher Scientific Baltics, Vilnius, Lithuania), and cloned into the pPICZα A vector (Invitrogen, Carlsbad, CA, USA), according to the manufacturer’s instructions. The resulting pPICZαA/GH2βGalS3*II recombinant plasmid was linearized with the *Mss*I (*Pme*I) restriction enzyme and transformed into *K. phaffii* X-33 cells (Invitrogen, Carlsbad, CA, USA) by electroporation (1500 V, 5 ms, expotential decay pulse) using the Gene Pulser Xcell Electroporation System (Bio-Rad, Hercules, CA, USA).

The *bgal42* gene encoding GH42 β-d-galactosidase was amplified using forward primer FS3mBgalXho 5′ CTGA**CTCGAG**AAAAGA**ATGCCGTCCCCGATCCCCGAGAAATC** 3′ and reverse primer RS3mBgalNot 5′ GACT**GCGGCCGC****TTAGGACTCGGCGATGACGGCTACGGCA** 3′, containing *Xho*I and *Not*I recognition sites, respectively. The PCR was performed using DNA polymerase *Hypernova* (Blirt, Gdańsk, Poland) and pBAD-GH42BgalS3* recombinant plasmid as a template. The PCR product of 2080 bp was purified from the reaction mixture, digested with *Xho*I and *Not*I endonucleases (Thermo Fisher Scientific Baltics, Vilnius, Lithuania), and cloned into the pPICZα A vector (Invitrogen, Carlsbad, CA, USA), digested with the same restriction enzymes. The resulting pPICZαA/GH42βGalS3*I recombinant plasmid was linearized with *Mss*I (*Pme*I) enzyme and transformed into *K. phaffii* X-33 cells (Invitrogen, Carlsbad, CA, USA) by electroporation.

The recombinant *K. phaffii* strains were selected on the YPDS (2% peptone K, 1% yeast extract, 2% glucose, 1 M sorbitol, 2% bacteriological agar) medium containing Zeocin (100 µg mL^−1^).

### 4.6. Production of Arthrobacter sp. S3* β-d-Galactosidases in the E. coli and Purification of Recombinant Enzymes

The production of *Arthrobacter* sp. S3* Bgal2 and Bgal42 enzymes was performed in *E. coli* LMG194 cells carrying the pBAD/Myc-HisA-GH2β-galS3* plasmid and pBAD-GH42BgalS3* plasmid, respectively. The cells were grown for about 18 h at 37 °C in LB medium (1% NaCl, 1% peptone K, 0.5% yeast extract) supplemented with 100 μg mL^−1^ ampicillin. Then, 20 mL of each culture was added to 1 L of LB medium containing 100 μg mL^−1^ of antibiotic and cultivated at 30 °C for 2.5 h with an agitation of 160 rpm until an OD_600_ of about 0.5 was met. A 20% solution of L-arabinose was then added to the final concentration of 0.02% and cultivation was continued for about 15 h until an OD_600_ of about 4 was met. Both cultures were then centrifuged (6000× *g*, 15 min, 4 °C), cell pellets were resuspended in 50 mL of buffer A (20 mM of potassium phosphate buffer at pH 6.0 containing 50 mM of KCl), and the cells were disrupted by sonication (5 cycles of 30 s with 1 min intervals at a vibration amplitude of 5 μm).

After centrifugation (12,000× *g*, 30 min, 4 °C), each supernatant was applied onto a 60 mL column with the weak anion exchanger Fractogel EMD DEAE (Merck, Darmstadt, Germany) equilibrated with buffer A. The column was washed with three volumes of buffer A and the recombinant enzyme (Bgal2 or Bgal42) was eluted with a linear gradient of potassium chloride from 50 to 1050 mM in the same buffer (four volumes of the column). Fractions exhibiting β-d-galactosidase activity were pooled, dialyzed against buffer A and loaded onto a Fractogel EMD TMAE strong anion exchanger (Merck, Darmstadt, Germany) (40 mL column) equilibrated with buffer A. The column was washed with two volumes of buffer A and the elution was performed with a linear gradient of potassium chloride from 50 to 850 mM in the same buffer (four volumes of the column). Fractions containing the recombinant enzyme (Bgal2 or Bgal42) were pooled, dialyzed against 20 mM of potassium phosphate buffer at pH 7.5 containing 150 mM of KCl and loaded onto a Superdex^TM^ 200 10/300 GL column (GE Healthcare Life Sciences, Uppsala, Sweden). The elution was performed using the same buffer. The purified *Arthrobacter* sp. S3* GH42 β-d-galactosidase (Bgal42) was dialyzed against 40 mM of potassium phosphate buffer at pH 7.0, and the purified GH2 β-d-galactosidase (Bgal2) was dialyzed against 40 mM of potassium phosphate buffer at pH 7.5. Proteins were then concentrated using the Amicon Ultra-15 30K Centrifugal Filter Device (Merck Millipore, Tullagreen, Carrigtwohill, Co Cork, Ireland). Afterwards, glycerol was added to a final concentration of 50% and the enzymes were stored at −20 °C. The concentration of proteins was determined using a Bradford Reagent and bovine serum albumin (BSA) as a standard (Sigma, St. Louis, MO, USA) [75].

The molecular weights of the native Bgal2 and Bgal42 enzymes were estimated by gel filtration (GF) using a Superdex^TM^ 200 10/300 GL column (GE Healthcare Life Science, Uppsala, Sweden) and a MW-GF-1000 Kit for Molecular Weights 29,000–700,000, purchased from Sigma (St. Louis, MO, USA).

### 4.7. Production of Arthrobacter sp. S3* β-d-Galactosidases in the K. phaffii

The *K. phaffii* X-33 cells transformed with pPICZαA/GH2βGalS3*II and pPICZαA/GH42βGalS3*I plasmids were grown in 25 mL of BMGY medium (2% peptone K, 1% yeast extract, 100 mM of potassium phosphate buffer at pH 6.0, 1.34% Yeast Nitrogen Base, 4 × 10^−5^% biotin, 1% glycerol) for 18 h at 30 °C with agitation (250 rpm). The cells were then centrifuged at 3000× *g* for 5 min, and th ecell pellets were resuspended to an OD_600_ of 1.0 in 50 mL of BMMY medium (2% peptone K, 1% yeast extract, 100 mM of potassium phosphate buffer at pH 6.0, 1.34% YNB, 4 × 10^−5^% biotin, 0.5% methanol). The recombinant yeast strains were then cultivated for 5 days at 25 °C with agitation (250 rpm). Cultures were supplemented with methanol every 24 h to a final concentration of 0.5%. Samples were collected every 24 h, centrifuged at 9600× *g* and the β-d-galactosidase activity in the post-culture fluid was measured with ONPG as a substrate. The reaction mixture consisted of 800 µL of ONPG (1 mg mL^−1^) and 200 µL of post-culture fluid. Hydrolysis was stopped by adding 300 µL of 1 M sodium carbonate, and absorbance was measured at 405 nm.

After 4 days of expression, the yeast cultures were centrifuged, the cell pellets were resuspended in the buffer A (20 mM of potassium phosphate buffer at pH 6.0 containing 50 mM of KCl), and the cells were disrupted by sonication (5 cycles of 1 min with 1 min intervals at a vibration amplitude of 5 μm). After centrifugation, the β-d-galactosidase activity in cell-free extracts was measured with ONPG.

### 4.8. Characterization of Arthrobacter sp. S3* Bgal2 and Bgal42 Hydrolytic Activity

The activity of the purified *Arthrobacter* sp. S3* Bgal2 and Bgal42 enzymes was determined with ONPG as a substrate. One unit of β-d-galactosidase activity was defined as being the quantity of enzyme releasing 1 μmol of *o*-nitophenol (*o*NP) per minute under optimum conditions, namely 22 °C and pH 7.5 for Bgal2 or 40 °C and pH 7.0 for Bgal42.

For pH activity studies, the hydrolysis of the synthetic substrate ONPG at a concentration of 1 mg mL^−1^ was performed in 20 mM of citrate-sodium phosphate buffer at pH 4.0 to 6.0, 20 mM of potassium phosphate buffer at pH 6.0 to 8.0, 20 mM of Tris-HCl buffer at pH 8.0–9.0 and 20 mM of glycine-NaOH buffer at pH 9.0 to 10.0 at room temperature (21–22 °C) for 5 min in the case of Bgal2 and 10 min in the case of Bgal42. The hydrolysis was halted by the addition of 1.5 M of Na_2_CO_3_ to the final concentration of 450 mM and the absorbance was measured at 405 nm.

To determine the effect of temperature on the *Arthrobacter* sp. S3* Bgal2 activity, the enzyme was incubated in 20 mM of potassium phosphate buffer at pH 7.5 containing 50 mM of KCl and 1 mg mL^−1^ of ONPG at 5 to 55 °C for 5 min. The hydrolysis was halted by heating the reaction mixtures at 95 °C for 5 min and the absorbance was measured at 405 nm. The *Arthrobacter* sp. S3* Bgal42 was incubated in 20 mM of potassium phosphate buffer at pH 7.0 containing ONPG at 5 to 60 °C for 10 min.

For the thermal stability studies, the Bgal2 and Bgal42 were incubated at various temperatures for different periods of time and the residual β-d-galactosidase activity was then measured under optimal conditions for each enzyme.

The effects of EDTA and various divalent metal ions on the recombinant GH2 β-d-galactosidase activity were measured by assaying the Bgal2 in 20 mM of HEPES-NaOH at pH 7.5, containing 5 mM of EDTA, MgCl_2_·6H_2_O, MnCl_2_·4H_2_O, CaCl_2_·2H_2_O, NiCl_2_·6H_2_O, CoCl_2_·6H_2_O and ZnCl_2_ at 22 °C for 5 min. The hydrolysis of ONPG was halted by heating at 95 °C for 5 min and the absorbance of the reaction mixtures was measured at 405 nm.

The effects of selected reducing agents on the Bgla2 activity were measured by assaying the β-d-galactosidase in 20 mM of potassium phosphate buffer with 50 mM of KCl (pH 7.5) containing 10 mM of DTT, glutathione, cysteine or TCEP at 22 °C for 5 min. The ONPG hydrolysis was halted by the addition of sodium carbonate to the final concentration of 450 mM and the absorbance was measured at 405 nm.

In the case of *Arthrobacter* sp. S3* Bgal42, 20 mM of HEPES-NaOH at pH 7.0 or 20 mM of potassium phosphate buffer at pH 7.0 were used, and the reactions were conducted at 40 °C.

Substrate specificity was estimated using PNPG, *p*NP-α-d-galactopyranoside, *p*NP-β-d-glucopyranoside, *p*NP-α-d-glucopyranoside, *p*NP-β-d-glucuronide, *p*NP-β-d-fucopyranoside, *p*NP-β-l-arabinopyranoside, *p*NP-β-d-xylopyranoside, *p*NP-β-d-mannopyranoside and *p*NP-β-d-cellobioside at pH 7.0 and 22 °C for Bgal2 or at 40 °C for Bgal42. The hydrolysis of the substrates was stopped after 5 or 10 min by the addition of sodium carbonate.

To determine the kinetic parameters of the recombinant *Arthrobacter* sp. S3* Bgal2, the hydrolysis of ONPG and lactose was performed in 20 mM of potassium phosphate buffer with 50 mM of KCl (pH 7.5) containing 1–5 mM substrates at 10 and 20 °C. The kinetic parameters of recombinant Bgal42 were determined in 20 mM of potassium phosphate buffer at pH 7.0, supplemented with ONPG and lactose at 10, 20, 30 and 40 °C. To estimate the amount of d-glucose released during lactose hydrolysis, the Glucose (GO) Assay Kit (Sigma, St. Louis, MO, USA) was used according to the manufacturer’s instructions. The *K*_m_ and *V*_max_ values were obtained using the Lineweaver–Burk equation.

The effects of d-glucose and d-galactose on the *Arthrobacter* sp. S3* Bgal42 activity were measured by assaying the enzyme in 20 mM of potassium phosphate buffer containing 1–5 mM of ONPG and 5 mM of monosaccharide at pH 7.0 and 30 °C for 1–5 min. The hydrolysis of ONPG was halted by the addition of sodium carbonate to the final concentration of 450 mM and the absorbance was measured at 405 nm. The effects of monosaccharides on the Bgal2 activity were estimated using 5–100 mM of d-glucose and d-galactose in 20 mM of potassium phosphate buffer with 50 mM of KCl (pH 7.5) containing 1–5 mM of ONPG as a substrate at 20 °C.

The hydrolysis of lactose in milk by the *Arthrobacter* sp. S3* Bgal2 and Bgal42 enzymes was performed using from 1 to 20 U of β-d-galactosidases per 1 mL of UHT milk (2% fat). The reaction mixtures were incubated at 10 °C for 24 h. After each 2 h period for 12 h and after 24 h, 1 mL samples were collected and the hydrolysis of lactose was halted by the addition of 17 µL of 20% sulphuric acid. The samples were then centrifuged (10,000× *g*, 30 min, 4 °C) to remove denatured proteins. The quantities of lactose, D-glucose and D-galactose were determined by HPLC using an Aminex HPX-87H column (Bio-Rad, Hercules, CA, USA), 5 mM of sulphuric acid as a mobile phase, and the Agilent 1200 Series chromatograph with Refractive Index Detector (Agilent Technologies, Santa Clara, CA, USA).

### 4.9. Characterization of Arthrobacter sp. S3* Bgal2 and Bgal42 Transglycosylation Activity

The synthesis of GOS was performed as described in our previous study [34], with some modifications. Reaction mixtures containing 10 or 20 U mL^−1^ of *Arthrobacter* sp. S3* Bgal42 in 20 mM of potassium phosphate buffer at pH 7.0 with 29–584 mM of lactose were incubated at 10, 30 and 40 °C for 24 h. Every 2 h for the first 10 h and after 24 h, 100 μL samples were collected and incubated at 95 °C for 5 min to inactivate the enzyme. The transglycosylation products were then analyzed by TLC. In the case of Bgal2, reaction mixtures containing 1 or 2 U mL^−1^ of the enzyme in 20 mM of potassium phosphate buffer at pH 7.5 with 50 mM of KCl and 29–584 mM of lactose were incubated at 10, 20 and 30 °C.

Lactulose was synthesized using 1 and 2 U of the *Arthrobacter* sp. S3* Bgal2 or 10 and 20 U of the Bgal42 per 1 mL of appropriate potassium phosphate buffer supplemented with 29–438 mM of lactose and equimolar concentrations of d-fructose. The reaction mixtures were incubated at 15 and 30 °C for Bgal2 and Bgal42, respectively. The reaction products were separated by TLC and detected as described previously [42].

Galactosyl-xylose and galactosyl-arabinose were synthesized at 15 °C using 1 and 2 U of the *Arthrobacter* sp. S3* Bgal2 per 1 mL of the buffered lactose solutions supplemented with D-xylose and L-arabinose, respectively.

The synthesis of alkyl galactopyranosides (1-butyl-, 1-hexyl-, 1-heptyl- and 1-octyl-) and galactosylated salicin catalyzed by the Bgal2 was performed under standard conditions (pH 7.5, 15 °C), and the reaction products were analyzed by TLC [42].

## 5. Conclusions

The GH family 2 β-D-galactosidase from psychrotolerant *Arthrobacter* sp. S3* that was obtained and characterized in this study has properties that make it an attractive biocatalyst in the food industry. It can be used both for the production of low-lactose milk under refrigeration conditions and for the synthesis of galacto- and heterooligosaccharides with prebiotic properties. The optimization of the conditions of the milk lactose digestion process should allow for the production of a health-promoting milk with a reduced lactose content that is simultaneously enriched with GOS, because the *Arthrobacter* sp. S3* Bgal2 shows both activities at 146 mM of lactose, which corresponds to the concentration of this disaccharide in milk. Moreover, the enzyme catalyzes the transfer of the galactosyl moiety from lactose to various chemical compounds at 15 °C; it could thus be an interesting biocatalyst for the production of cosmetics and pharmaceuticals, especially in the glycosylation of thermosensitive chemicals.

The advantages of the *Arthrobacter* sp. S3* Bgal42 enzyme include its relatively low molecular weight, which allows its secretion by *K. phaffii* cells, its high activity at pH 6, and its transglycosylation optimum at 30 °C (optimal growth conditions for *K. phaffii*), which should allow its application as a whole-cell biocatalyst for GOS synthesis in whey, after cloning the gene under the control of a constitutive *GAP* (glyceraldehyde-3-phosphate dehydrogenase) gene promoter.

## 6. Patents

The *Arthrobacter* sp. S3* GH2 β-d-galactosidase has been granted a Polish patent number Pat.231905.

## Figures and Tables

**Table 1:** Comparison of the amino acid sequence of *Arthrobacter* sp. S3* Bgal2 with amino acid sequences of cold-active GH2 β-d-galactosidases from bacteria of the *Arthrobacter* genus and *Escherichia coli* LacZ.

Organism	GenBank Accession No.	Identity (%)	Similarity (%)
*Arthrobacter* sp. ON14	ADJ18283.1	99.7	99.8
*Arthrobacter psychrolactophilus* F2	BAF33372.1	97.3	98.4
*Arthrobacter* sp. C2-2	CAD29775.1	84.6	93.6
*Arthrobacter* sp. B7	AAA69907.1	83.0	92.7
*Arthrobacter* sp. 20B	ACI41243.1	72.1	87.0
*Arthrobacter* sp. SB	AAQ19029.1	71.7	87.3
*Arthrobacter* sp. 32cB	AHY00656.1	46.3	69.2
*Escherichia coli* K12	ABN72582.1	34.1	59.5

A homology search was performed using the FASTA 1 program at the EMBL-EBI [26].

**Table 2:** Comparison of the amino acid sequence of *Arthrobacter* sp. S3* Bgal42 with amino acid sequences of cold-active β-d-galactosidases belonging to the GH family 42 and *Thermus thermophilus* β-d-galactosidase.

Organism	GenBank Accession No.	Identity (%)	Similarity (%)
*Arthrobacter* sp. ON14	ADJ18282.1	99.7	99.7
*Arthrobacter* sp. 32c	ACU00913.1	71.8	88.5
*Arthrobacter* sp. B7	AAA75601.1	48.2	67.9
*Carnobacterium maltaromaticum* BA	AAF16519.1	41.7	70.5
*Planococcus* sp. SOS Orange	AAF75984.1	39.8	69.3
*Planococcus* sp. L4	ABI64125.1	39.2	69.6
*Thermus thermophilus* A4	BAA28362.1	35.8	59.7
*Rahnella* sp. R3	AJC52391.1	35.3	51.1
*Marinomonas* sp. ef1	WP_100635792.1	29.3	44.0

A homology search was performed using the FASTA 1 program at the EMBL-EBI [26].

**Table 3:** Summary of the purification of recombinant *Arthrobacter* sp. S3* Bgal2 and Bgal42 β-d-galactosidases obtained from 1 L of *E. coli* LMG194 culture.

Purification Step	Protein (mg)		Total Activity (U)		Specific Activity (U mg^−1^)		Yield (%)	
	Bgal2	Bgal42	Bgal2	Bgal42	Bgal2	Bgal42	Bgal2	Bgal42
Cell-free extract	451	480	1580	3360	3.5	7.0	100	100
IEX, Fractogel EMD DEAE	48.0	52.0	1300	2830	27.1	54.4	82.3	84.2
IEX, Fractogel EMD TMAE	20.0	35.0	1100	2500	55.0	71.4	69.6	74.4
GF, Superdex^TM^ 200 10/300 GL	11.0	20.0	956	2310	86.9	115	60.5	68.7

The activity was measured with ONPG as a substrate.

**Table 4:** Effects of divalent metal ions and selected reagents on *Arthrobacter* sp. S3* Bgal2 and Bgal42 enzyme activity.

Metal Ion/EDTA (5 mM)	Relative Activity (%)		Reducing Agent (10 mM)	Relative Activity (%)	
	Bgal2	Bgal42		Bgal2	Bgal42
None	100	100	None	100	100
Mg^2+^	145 ± 4.6	199 ± 5.5	DTT	81 ± 2.7	102 ± 3.1
Ca^2+^	55 ± 1.7	176 ± 4.8	Cysteine	28 ± 1.1	91 ± 2.4
Mn^2+^	76 ± 2.5	72 ± 2.3	Glutathione	47 ± 1.5	51 ± 2.0
Co^2+^	9 ± 1.1	63 ± 2.1	TCEP	2 ± 0.7	1 ± 0.7
Ni^2+^	9 ± 1.0	44 ± 1.8			
Zn^2+^	0 ± 0.2	2 ± 0.8			
EDTA	15 ± 1.4	306 ± 8.9			

The activity was measured with ONPG as a substrate.

**Table 5:** Kinetic parameters of the ONPG and lactose hydrolysis catalyzed by *Arthrobacter* sp. S3* Bgal2 and Bgal42 enzymes.

Enzyme	Temperature (°C)	*K*_m_ (mM)		*V*_max_ (U mg^−1^)		*k*_cat_ (s^−1^)		*k*_cat_/*K*_m_ (s^−1^ mM^−1^)	
		ONPG	Lactose	ONPG	Lactose	ONPG	Lactose	ONPG	Lactose
Bgal2	10	0.71 ± 0.11	11.70 ± 0.22	32.13 ± 1.15	11.00 ± 0.35	59.94 ± 2.13	20.50 ± 1.12	83.92	1.75
	20	0.32 ± 0.07	16.70 ± 0.81	46.81 ± 1.54	21.42 ± 0.91	87.30 ± 2.47	39.91 ± 1.41	274.10	2.39
Bgal42	10	1.38 ± 0.23	1.17 ± 0.25	73.90 ± 1.78	0.27 ± 0.09	91.70 ± 2.88	0.34 ± 0.13	66.45	0.29
	20	0.76 ± 0.04	0.92 ± 0.18	117.70 ± 2.01	0.48 ± 0.12	146.05 ± 3.15	0.60 ± 0.15	193.40	0.64
	30	1.23 ± 0.18	1.91 ± 0.33	276.94 ± 3.26	0.99 ± 0.18	343.75 ± 2.61	1.24 ± 0.17	278.49	0.65
	40	3.92 ± 0.74	1.53 ± 0.35	512.31 ± 5.13	0.99 ± 0.20	635.91 ± 5.89	1.24 ± 0.18	162.20	0.81

## Data Availability

The 16S rDNA, *bgal2* and *bgal42* gene sequences reported in this article have been deposited in the GenBank database at NCBI (National Center for Biotechnology Information) and assigned accession numbers PQ463689, PQ479351 and PQ479352, respectively. Other data can be obtained from the corresponding author upon reasonable request.

## Supplementary Materials

The following supporting information can be downloaded at: https://www.mdpi.com/article/10.3390/ijms252413354/s1.
