# Supplementary material for: Heterologous Production, Purification and Characterization of Two Cold-Active β-d-Galactosidases with Transglycosylation Activity from the Psychrotolerant Arctic Bacterium Arthrobacter sp. S3* Isolated from Spitsbergen Island Soil
Source: Int J Mol Sci. 2024 Dec 12;25(24):13354. doi: 10.3390/ijms252413354 (PMC11677235; doi:10.3390/ijms252413354)

Figure S1. The multiple sequence alignment of *Arthrobacter* sp. S3* Bgal2 (S3-Bgal2; GenBank Accession no. XKB78123) with GH2 β-D-galactosidases from psychrotolerant bacteria *Arthrobacter* sp. C2-2 (C2-2-Bgal; GenBank Accession no. CAD29775.1) and *Arthrobacter* sp. 32cB (32cB-Bgal; GenBank Accession no. AHY00656.1). Conserved regions are marked with asterisks, the catalytic residues are shaded red, the residues involved in the hydrolysis and transglycosylation are shaded green and cysteine residues are shaded yellow. The multiple sequence alignment was performed using Clustal Omega (1.2.4) program (https://www.ebi.ac.uk/jdispatcher/msa/clustalo last accessed on 30 AUG 2024).

```
32cB-Bgal       MSVETPSALADSSPHTAPGSAGRSLELGAADIQDLESFEAG-RGALPARAYLQSDAPRLS  59
S3-Bgal2        --------------------------MTPADVSYITDQGPGSGLRVPARSWLNSDAPSLS  34
C2-2-Bgal       --------------------------MTTADVSYLTDQGPGSGRRVPARSWLHSDAPALS  34
                                          :  **:. : .   *    :***::*:**** **

32cB-Bgal       LNGEWQFRLSPGSRVAPDDGWQLG--EA----------LNGFESLPVPSSWPMHGHG---  104
S3-Bgal2        LNGDWRFRLLPAAPGTPGAGSVLPDGESVEGVAAESFDDSAWDTLPVPSHWVLHGEGKYG  94
C2-2-Bgal       LNGDWRFRLLPAAPGTAGAGSVLPSGETVEGVAAESYDDAAWDTLPVPSHWVMGQDGKYG  94
                ***:*:*** *.:  : . *  *   *:            .:::***** * :  .*

32cB-Bgal       APAYTNVQFPFAVEPPHVPEANPIGDHLVVFEAGPEFFPH----ALLRFDGIESAGTVWL  160
S3-Bgal2        RPIYTNVQYPFPVDPPFVPDANPTGDYRRSFDVPAAWFESTTAALTLRFDGVESRYKVWV  154
C2-2-Bgal       RPIYTNVQYPFPIDPPHVPDANPTGDFRRRFDVPAQWFESTTAALTLRFDGVESRYKVWV  154
                 * *****:** ::**.**:*** **.   *:.   :*        *****:**  .**:

32cB-Bgal       NGVELGTTRGSRLAHEFDVSGILEQGENTLAVRVAQFSAASYVEDQDMWWLPGIFRDVTL  220
S3-Bgal2        NGQEIGVGSGSRLAQEFDVTAALRPGSNLLVVRVHQWSAASYLEDQDQWWLPGIFRDVTL  214
C2-2-Bgal       NGQEIGVGSGSRLAQEFDVSDALRAGSNLLVVRVHQWSAASYLEDQDQWWLPGIFRDVTL  214
                ** *:*.  *****:****:  *. *.* *.*** *:*****:**** ************

32cB-Bgal       QARPAAGIDDVFVHAGYDHITGEGI--------LKVEASRGGQAIDAVVRVPELALELAA  272
S3-Bgal2        AARPAGGITDVWLRTGWTADTGAGAGQGAGTGTLDPEIIAEADAYPVTLTVPALGVAVRW  274
C2-2-Bgal       QARPAGGITDAWLRTGWSARSG------AGTGTIDPEITADATAFPVTLSVPELGVNVTW  268
                 ****.** *.::::*:   :*           :. *    . *  ..: ** *.: :

32cB-Bgal       -----GTEVRVPAVEPWSAEVPKLYEAAVSAAGESVALQIGFRSIAIEDAQFKVNGRRIL  327
S3-Bgal2        DSPADVVPFTVDNVEPWSAELPRLYEATVSSAAESITLRLGFRTVEIVGDQFLVNGRRVV  334
C2-2-Bgal       KSAEEVAPLALENVEPWSAEVPRLYEASVSSAAESISVRLGFRTVRIVGDQFLVNGRRVV  328
                      . . :  *******:*:****:**:*.**:::::***:: * . ** *****::

32cB-Bgal       LRGVNRHEHHPRLGRVVPRDVVEAELRLMKQHNINAIRTSHYPPHPQFLALADQLGFYVV  387
S3-Bgal2        FHGVNRHETNPDRGRVFDEADAREDLALMKRFNVNAIRTSHYPPHPRLLDLADEMGFWVI  394
C2-2-Bgal       FHGVNRHETHPDRGRVFDEAGAREDLALMKRFNVNAIRTSHYPPHPRLLDLADEMGFWVI  388
                ::****** :*  ***. .  .. :* ***:.*:************::* ***::**:*:

32cB-Bgal       LECDLETHGFESAGWAQNPSDDPQWEDALVDRMRRTVERDKNHASVVMWSLGNEAGTGRN  447
S3-Bgal2        LESDLETHGFHRQGWVDNPSDVPAWRDAFVDRMERTVERDKNHASIVMWSLGNESGTGAN  454
C2-2-Bgal       LECDLETHGFEAGGWVENPSDVPAWRDALVDRMERTVERDKNHPSIVMWSLGNESGTGSN  448
                **.*******.  **.:**** * *.**:****.********* *:********:*** *

32cB-Bgal       LAAMSRWTKDRDPSRPIHYEGDWSSEHVDVYSRMYASQAETALIGQGIEPAL---NDAAL  504
S3-Bgal2        LAAMAAWTHARDASRPVHYEGDYTGAYTDVYSRMYSSIPETDSIGRNDSHALLLGCDAAE  514
C2-2-Bgal       LAAMAAWAHARDSSRPVHYEGDYTGAYTDVYSRMYSSIPETDSIGRNDSHALLLGCDSAE  508
                ****: *:: ** ***:*****:.  :.*******:*  **  **:. . **    *:*

32cB-Bgal       DARRRAMPFVLCEYVHAMGNGPGGMSEYQALFEKYPRLMGGFVWEWLEHGITVSTADGVD  564
S3-Bgal2        SARQRTRPFILCEYVHAMGNGPGAMDQYEELVDKYPRLHGGFVWEWRDHGIRANTADGTE  574
C2-2-Bgal       SARQRTKPFILCEYVHAMGNGPGAMDQYEALVDKYPRLHGGFVWEWRDHGIRTRTAEGME  568
                .**:*: **:*************.*.:*: *.:***** ******* :*** . **:* :

32cB-Bgal       HYGYGGDFGEEVHDGNFVTDGLVDADRRPRPGLLDFKKVIEPLRIDVARDWTGF---TLR  621
S3-Bgal2        FFAYGGDFGEVIHDSNFVMDGMVLSDSTPTPGLHEYKQIVSPIRLSLALDDGGAPRLTVA  634
C2-2-Bgal       FFAYGGDFGEVVHDSNFVMDGMVLSDSTPTPGLYEFKQIVSPIRLGLSLPAGGKPTLAVA  628
                .:.******* :**.*** **:* :*  * *** ::*:::.*:*:.::    *    ::

32cB-Bgal       NGQDFADTSAFSFRYEVEADGGALDGGTVDVAPVA----PQSETVVELPGSVAALAAGLS  677
S3-Bgal2        NLRHTADASDVVLRWRVEHNGAVAASGELAVDSADGPPRAGESVTLTLPPVA------AA  688
C2-2-Bgal       NLRHTADASDVVLRWRVEHDGAVAASGEVAAEGSDGPLRAGESATIALPAMP------AA  682
                * :. **:* . :*:.** :*..  .* : .          ....: **          :

32cB-Bgal       DGRPAVLTVRAVLGADSAWADAGHEVAWGQSVR-----EPGAPVPPAPVEPVQVQDSELT  732
S3-Bgal2        PEGETWLTMEVVLRDATEWAPAGHPLAATQLDLSAPQSAPRAPRPAAPVS----GAAPVE  744
C2-2-Bgal       PLGETWLTVEAVLRDATGWAPAGHPLGAVQLDLSAPAVPTRSPRPATPLD----GALPVS  738
                    : **:..**   : ** *** :.  *           :* * :*:.        :

32cB-Bgal       LGPVVFSRATGMPTSIGGVPVEKLGLTLWWAPTDNDLGREWGGAD-------------ER  779
S3-Bgal2        LGHAMFD--AGALVALAGLPVSGPRLELWRAPTDNDRGKGFGAYGPEDPWLNNGKGVPAP  802
C2-2-Bgal       LGPATFD--AGTLVSLAGQPVSGPRLELWRAPTDNDRGAGFGAYGPGDPWLNSGRGVPAP  796
                ** . *.  :*  .::.* **.   * ** ****** *  :*. .

32cB-Bgal       PLATQWKDAGLNRLHTRLLGISANPGQDGGETLTVRTRVSAADKQYGVLVDYTWSTDGET  839
S3-Bgal2        SSEAVWKQAGLDRLTRRVEDVAALPD-----GLRVRTRYAAANSAASVAVEENWQLAGEE  857
C2-2-Bgal       SSEAVWKQAGLDRLTRRVEDVAALPD-----GIRVRTRYAAADSTHSVAVEENWQLDGGE  851
                   : **:***:**  *: .::* *.      : **** :**:.  .* *: .*.  *

32cB-Bgal       VGLRTQVRRDGTWVNRGFEVEWARIGLEFVLGEETELVSWFGQGPHQSYPDTGQGARAGW  899
S3-Bgal2        LWLRLDIMPS-----AGWDMVFPRIGVRLDLPTDIDGASWFGAGPRESYADSMHATVVGR  912
C2-2-Bgal       LCLRIDITPS-----AGWNLVWPRIGVRWDLPTDVDGAAWFGAGPRESYPDSMHATVVAR  906
                : ** ::  .      *::: : ***:.  * :  : .:*** **::** *: :.: ..

32cB-Bgal       FSLPLAKMDVEYVRPQECGARSGSRSAALQLGGR-TLEICGDPFALTVRP------YSQD  952
S3-Bgal2        HSGSIDELNVPYARPQETGHRSDVRSLELSRNGAPWLRIEADPDALGRRPGFSLARHTAQ  972
C2-2-Bgal       HAASLEELNVPYARPQETGHRSDVRWLELDRAGAPWLRIDAEPDAAGRRPGFSLARHTAQ  966
                .:  : :::* *.**** * **. *   *.  *   *.* .:* *   **      :: :

32cB-Bgal       VLDAAAHRPDLKADGRTYLYVDHALRGVGTAACGPGVLEQYRLKPRDADFILTLKVRS  1010
S3-Bgal2        QVTAAGHPHELPASSHSYLYLDAAQHGLGSRACGPDVWPDFALRPEARTLVLRFSAL-  1029
C2-2-Bgal       EIAAAGHPHELPTPSHSYLYVDAAQHGLGSRACGPDVWPDFALRPEARTLKLRISPA-  1023
                 : **.*  :* : .::***:* * :*:*: ****.*  :: *:*.   : * :.
```

Figure S2. The multiple sequence alignment of *Arthrobacter* sp. S3* Bgal42 (S3-Bgal42; GenBank Accession no. XKB78124) with GH42 β-D-galactosidases from psychrotolerant bacteria *Arthrobacter* sp. 32c (32c-Bgal; GenBank Accession no. ACU00913.1) and *Rahnella* sp. R3 (RR3-Bgal; GenBank Accession no. AJC52391.1), and thermophilic *T. thermophilus* A4 (TT-Bgal; GenBank Accession no. BAA28362.1). The catalytic residues are shaded red, the residues involved in D-galactose binding are shaded green and cysteine residues are shaded yellow. The multiple sequence alignment was performed using Clustal Omega (1.2.4) program (https://www.ebi.ac.uk/jdispatcher/msa/clustalo last accessed on 30 AUG 2024).

```
RR3-Bgal    ----------------------------MTKFPLLSSKISGLLHGADYNPEQWLDHPDVL   32
TT-Bgal     ------------------------------------------MLGVCYYPEHWPK--ERW   16
S3-Bgal42   ----------MPSPI-PEKSSRPDIAASELRTTRLWESLPGLSYGGDYNPEQWSR--DIR   47
32c-Bgal    MGKRFPSGWFSPRVHPPRRQRSPMTNQATPGTASVWNNIEGIGFGGDYNPEQWPV--SVR   58
                                                        *  * **:*    .

RR3-Bgal    VRDVEMMKEARCNVMSVGIFSWSALEPEEGRYTFDWMDQVLNRLHENGISVFLATPSGAR   92
TT-Bgal     KEDARRMREAGLSHVRIGEFAWALLEPEPGRLEWGWLDEAIATLAAEGLKVVLGTPTATP   76
S3-Bgal42   LEDIQLMQQAGVNALSVGIFSWSWLEPSEGQYDFDWLDEIMDNLAAAGIKVALATATAAP   107
32c-Bgal    LEDLELMQEAGVNFLSVGIFSWALLEPAEGQYDFGWLDDVMDNLHGIGVKVALATATAAP   118
             .* . *::*  . : :* *:*: ***  *:  :.*:*: :  *   *:.* *.* :.:

RR3-Bgal    PAWMSQKYPQVLRVGRDRVPALHGGRHNHCMSSPVYREKVQLMNGQLAKRYAHHPAVIGW   152
TT-Bgal     PKWLVDRYPEILPVDREGRRRRFGGRRHYCFSSPVYREEARRIVTLLAERYGGLEAVAGF   136
S3-Bgal42   PAWLVHKHPEILPVTADGTVLGQGSRRHYSPSSSIYRQYAAKITRTLAERYKGHPALVLW   167
32c-Bgal    PAWLVRKHPEILPVTADGTTLGPGSRRHYTPSSAVYRKYAAGITRVLAERYKDHPALALW   178
            * *:  ::*::* *  :      *.*:::  ** :**: .  :   **:**    *:  :

RR3-Bgal    HISNEYGG----ECHCDTCQGQFRDWLKARYVTLDALNKAWWSTFWSHTYTDWSQLESPS   208
TT-Bgal     QTDNEYGCHDTVRCYCPRCQEAFRGWLEARYGTIEALNEAWGTAFWSQRYRSFAEVELPH   196
S3-Bgal42   HVDNELGCHVD-QFHGPEDAAAFRSWLARRYGSIEALNEAWGTAFWSQRYGNFEEIFTPS   226
32c-Bgal    HVDNELGCHVS-EFYGEEDAAAFRLWLERRYGTIDALNAAWGTAFWSQHYGSFEEILPPG   237
            : .** *     . :       ** **  ** :::*** ** ::***: * .: ::  *

RR3-Bgal    PQGENGVHGLNLDWRRFNTDQVTRFCSEEIRPLKAENPALPATTNFMEYF--NDYDYWKL   266
TT-Bgal     LTVAEPNPSHLLDYYRFASDQVRAFNRLQVEILRAHAPGKFVTHNFMGFF--TDLDAFAL   254
S3-Bgal42   IAPTTLNPTQQLDFARFNSWALIDFYDMLVAVIREVTPHIPTTTNFMASSATKALDYFDW   286
32c-Bgal    VAPSTLNPGQQLDFQRFNSWALMDYYRSLVAVLREVTPAVPCTTNLMASSATKSMDYFSW   297
                       **: ** :  :  :    :  ::   *    * *:*     .  * :

RR3-Bgal    AGVLDFISWDSYPMWHTR-----------QDDIGLAAYTAMYHDLMRTLKQGKPFVLMES   315
TT-Bgal     AQDLDFASWDSYPLGFTDLMPLPPEEKLRYARTGHPDVAAFHHDLYRGVGRGRFWV-MEQ   313
S3-Bgal42   AEHVDVVANDHYLVA---------------SDTERHIELAFSADLTRGVAGGKPWMLMEH   331
32c-Bgal    AKDLDVIANDHYLVA---------------ADPERHIELAFSADLTRGIAGGDPWILMEH   342
            *  :*. : * * :                         *:  ** * :  *  ::  **

RR3-Bgal    TPSFTNWQPTSKLKKPGMHILSSLQAVAHGADSVQYFQWRKSRGSCEKFHGAVVDHVGHI   375
TT-Bgal     QPGPVNWAPHNPSPAPGMVRLWTWEALAHGAEVVSYFRWRQAPFAQEQMHAGLHRPDS-A   372
S3-Bgal42   STSAVNWQPRNMPKLPGEMHRNSLAHVARGADAVMFFQWRQSRAGSEKFHSAMVPHSG-T   390
32c-Bgal    STSAVNWQPRNQPKMPGEMLRNSLAHVARGADAVMFFQWRQSFAGSEKFHSAMVPHGG-R   401
              . .** * .    **     :   :*:**: * :*:**::  . *::*..:    .

RR3-Bgal    DTRVGREVAELGSILSALAPVAGSRVEAKVAIIFDWESRWAMDDAMGPRNAGLHYENTVA   435
TT-Bgal     PDQGFFEAKRVAEELAAL--ALPPVAQAPVALVFDYEAAWIYEVQPQ--GAEWSYLGLVY   428
S3-Bgal42   NTRVWREVVELGAALQAMAPVQGSLVESRTAIVFDYESWWASELDSHP-STDVKYLELLR   449
32c-Bgal    DTRVWREVVDLGAALQLLAPVRGSRVESRAAIVFDYEAWWASEIDSKP-SIDVRYLDLLR   460
              :   *.  :.  *  :  .    .:: .*::**:*: *  :      .    *   :

RR3-Bgal    DHYRALWAQGIAVDVINADCDLQGYDLVIAPMLYMVREGVGERISAFVQAGGRFVATYWS   495
TT-Bgal     LFYSALRRLGLDVDVVPPGASLRGYAFAVVPSLPIVREEALEAFRE---AEGPVLFGPRS   485
S3-Bgal42   AFHRSLFLRGITTDFVHPSANLDGYDLILACTLYSVNDADAANIAAAATRGATVLVSYFS   509
32c-Bgal    AFHRSLFLRGVSVDMVHPSASLDGYDLVLVCTLYSVTDEAAANIAAAAAGGATVLVSYFS   520
             .: :*   *: .*.:  ...* ** : :.  *  * :     :       . .:    *

RR3-Bgal    GIVNETDLCFLNGFPGPLRPVLGIWAEEIDSLTDEQHNSVAGVEGNALGLSGPYRASQLC   555
TT-Bgal     GSKTETFQIPKELPPGPLQALLPLKVVRVESLPPG-LLE------VAEGALGRFPLGLWR   538
S3-Bgal42   GIVDENDAVRLGGYPGAFRDLLGISVEEFHPLPESGSVT------VDAGWS----GTIWS   559
32c-Bgal    GITDEKDHVRLGGYPGAFRELLGVRVEEFHPLLAGSQLK------LSDGTV----SSIWS   570
            *   *.        ** :: :* : . ... *                *

RR3-Bgal    EVIHLEGAAALATYGDDFYAGNPAVTVNLYGKGQAYYVASRNDQQFHADFFTALAKEMKL   615
TT-Bgal     EWVEAPLK---------PLLTFQDGKGALYREGRYLYLAAWPSPELAGRLLSALAAEAGL   589
S3-Bgal42   EHVHLAGAEALARFTEYPLAGVPALTRNTAGTGSAWYLATFPDAESLDGIVGTLLEESGV   619
32c-Bgal    EHVHLDGAEAFQTFTGYPLEGVPSLTRRAVGTGAAWYLATFPDRDGIESLVDRLLAESGV   630
            * :.                     .      *   *:*:  . :    :.  *  *  :

RR3-Bgal    PRAINTPLPEGVTAARR-TDGESEFIFLQNYNADNQTVALPQDYQDIVHGGNLPRKLTLP   674
TT-Bgal     KVLS--L-PEGLRLRR-----RGTWVFAFNYGPEAVEAPASEGA-RFLLGSRRVGPYD--   638
S3-Bgal42   VAPA--IAAPGVELTRRRAADGTGYLFAVNHGASDAAVAA-SGI-ELISDTPFAGVVAAG   675
32c-Bgal    SPVA--EADAGVELTRRRSADGGSFLFAINHTRAAASVRA-SGT-DVLSGERFTGTVEAG   686
                      *:   *        ::*  *:      .    ..  .: .

RR3-Bgal    AFGCQILTRKITQ        687
TT-Bgal     ---LAVWEEA---        645
S3-Bgal42   --AVAVIAES---        683
32c-Bgal    --SVAVIAED---        694
                 :  .
```

Figure S3. The TLC analysis of lactulose synthesis catalyzed by the *Arthrobacter* sp. S3* Bgal42 enzyme. Reaction mixtures containing 10 U mL^{-1} (**A**) and 20 U mL^{-1} (**B**) *Arthrobacter* sp. S3* GH42 β-D-galactosidase and equimolar amounts of lactose and D-fructose as substrates were incubated at 30 °C for 6 h. Lane 1 – D-galactose, lane 2 – D-glucose, lane 3 – D-fructose, lane 4 – lactose, lane 5 – lactulose, lane 6 – 29 mM concentration of each substrate in the reaction mixture, lane 7 - 58 mM concentration of each substrate in the reaction mixture, lane 8 - 146 mM concentration of each substrate in the reaction mixture, lane 9 - 292 mM concentration of each substrate in the reaction mixture, lane 10 - 438 mM concentration of each substrate in the reaction mixture.

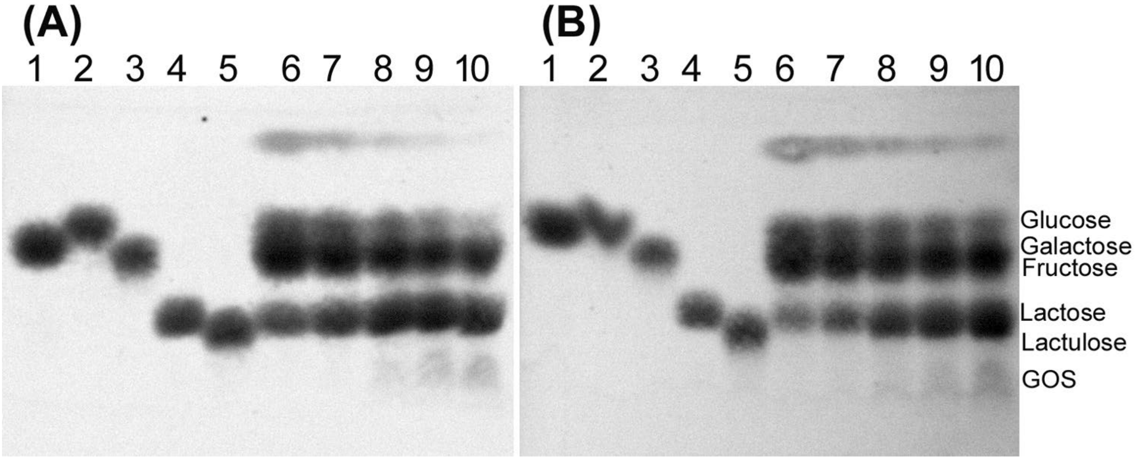

Figure S4. The HPLC analysis of lactose (**A**) and lactulose (**B**) hydrolysis catalyzed by the *Arthrobacter* sp. S3* Bgal42 enzyme. Reaction mixtures containing 35 U mL^{-1} *Arthrobacter* sp. GH42 β-D-galactosidase and 120 mM disaccharides as substrates in 20 mM potassium phosphate buffer (pH 7.0) were incubated at 10 °C for 24 h and then separated on the Aminex HPX-87H column. Glu, D-glucose; Gal, D-galactose; Fru, D-fructose.

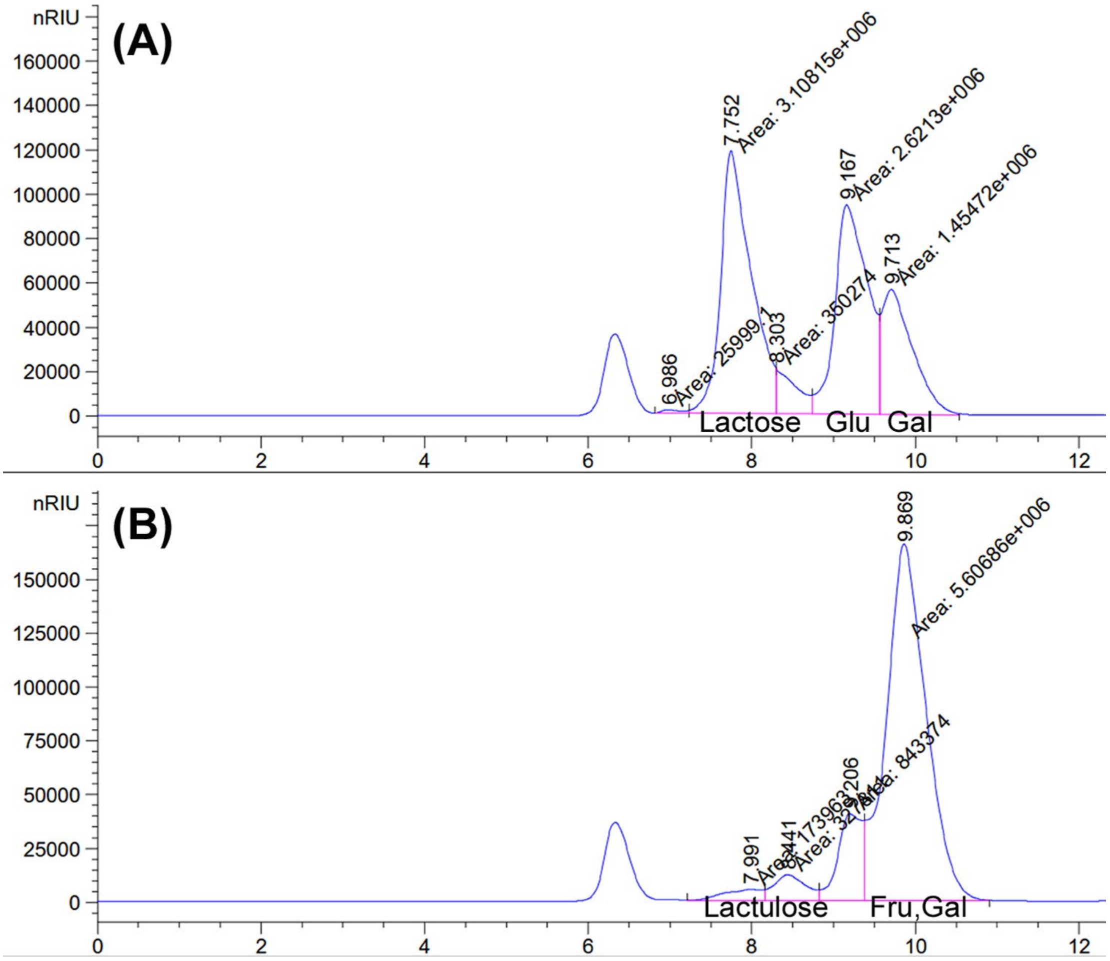

Figure S5. The HPLC analysis of lactulose hydrolysis catalyzed by the *Arthrobacter* sp. S3* Bgal2 enzyme. The reaction mixture containing 8 U mL^{-1} *Arthrobacter* sp. GH2 β-D-galactosidase and 120 mM disaccharide as a substrate in 20 mM potassium phosphate buffer with 50 mM KCl (pH 7.5) was incubated at 10 °C for 24 h and then separated on the Aminex HPX-87H column. Gal, D-galactose; Fru, D-fructose; OS, oligosaccharides.

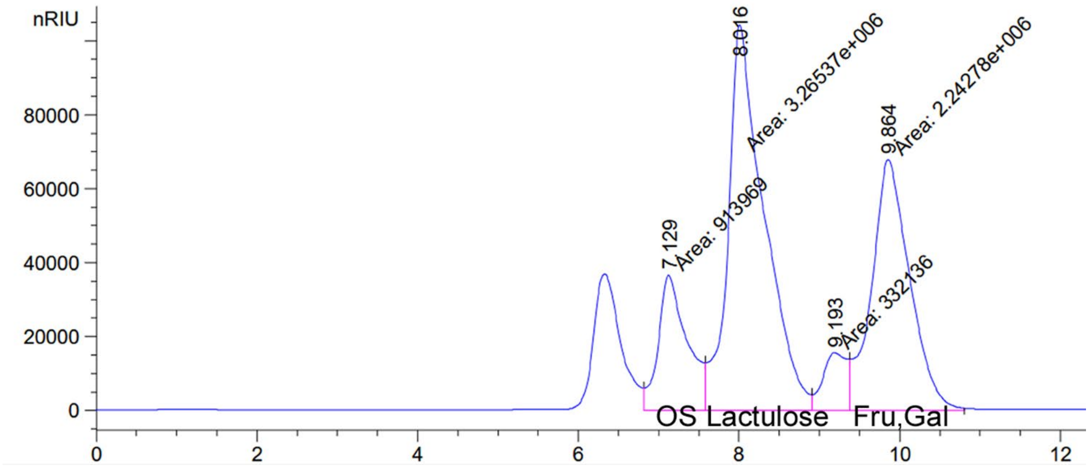

Figure S6. The scheme of recombinant pBAD/*Myc*-HisA-GH2β-galS3* plasmid construction.

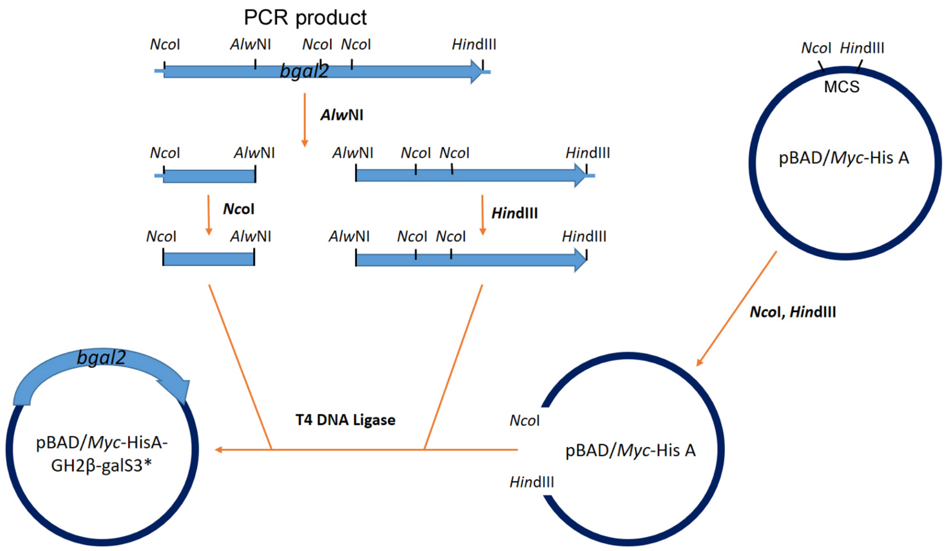

Figure S7. The scheme of recombinant pBAD-GH42BgalS3* plasmid construction.

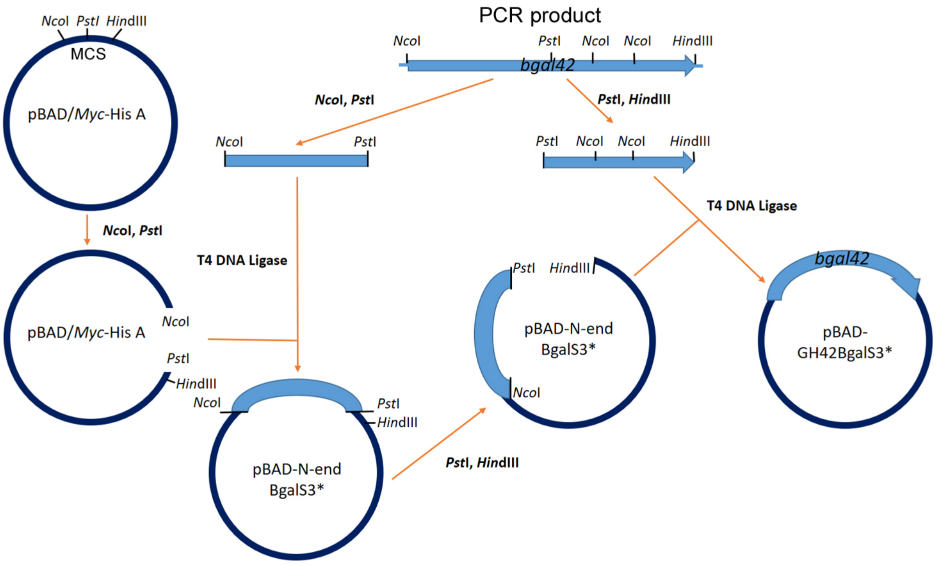

Supplement: Supplementary file 1 [file ijms-25-13354-s001.zip › ijms-3345264-supplementary.pdf]
